# Investigating Measurement Equivalence of Smartphone Sensor–Based Assessments: Remote, Digital, Bring-Your-Own-Device Study

**DOI:** 10.2196/63090

**Published:** 2025-04-03

**Authors:** Lito Kriara, Frank Dondelinger, Luca Capezzuto, Corrado Bernasconi, Florian Lipsmeier, Adriano Galati, Michael Lindemann

**Affiliations:** 1 F. Hoffmann-La Roche Ltd Basel Switzerland

**Keywords:** Floodlight Open, multiple sclerosis, smartphone, sensors, mobile phone, wearable electronic devices, digital health, equivalence, device equivalence, cognition, gait, upper extremity function, hand motor function, balance, digital biomarker, variability, mHealth, mobile health, autoimmune disease, motor, digital assessment

## Abstract

**Background:**

Floodlight Open is a global, open-access, fully remote, digital-only study designed to understand the drivers and barriers in deployment and persistence of use of a smartphone app for measuring functional impairment in a naturalistic setting and broad study population.

**Objective:**

This study aims to assess measurement equivalence properties of the Floodlight Open app across operating system (OS) platforms, OS versions, and smartphone device models.

**Methods:**

Floodlight Open enrolled adult participants with and without self-declared multiple sclerosis (MS). The study used the Floodlight Open app, a “bring-your-own-device” (BYOD) solution that remotely measured MS-related functional ability via smartphone sensor–based active tests. Measurement equivalence was assessed in all evaluable participants by comparing the performance on the 6 active tests (ie, tests requiring active input from the user) included in the app across OS platforms (iOS vs Android), OS versions (iOS versions 11-15 and separately Android versions 8-10; comparing each OS version with the other OS versions pooled together), and device models (comparing each device model with all remaining device models pooled together). The tests in scope were Information Processing Speed, Information Processing Speed Digit-Digit (measuring reaction speed), Pinching Test (PT), Static Balance Test, U-Turn Test, and 2-Minute Walk Test. Group differences were assessed by permutation test for the mean difference after adjusting for age, sex, and self-declared MS disease status.

**Results:**

Overall, 1976 participants using 206 different device models were included in the analysis. Differences in test performance between subgroups were very small or small, with percent differences generally being ≤5% on the Information Processing Speed, Information Processing Speed Digit-Digit, U-Turn Test, and 2-Minute Walk Test; <20% on the PT; and <30% on the Static Balance Test. No statistically significant differences were observed between OS platforms other than on the PT (*P*<.001). Similarly, differences across iOS or Android versions were nonsignificant after correcting for multiple comparisons using false discovery rate correction (all adjusted *P*>.05). Comparing the different device models revealed a statistically significant difference only on the PT for 4 out of 17 models (adjusted *P*≤.001-.03).

**Conclusions:**

Consistent with the hypothesis that smartphone sensor–based measurements obtained with different devices are equivalent, this study showed no evidence of a systematic lack of measurement equivalence across OS platforms, OS versions, and device models on 6 active tests included in the Floodlight Open app. These results are compatible with the use of smartphone-based tests in a bring-your-own-device setting, but more formal tests of equivalence would be needed.

## Introduction

Multiple sclerosis (MS) is a chronic, demyelinating autoimmune disease of the central nervous system, which can manifest in functional impairment in cognitive and motor abilities, and the subsequent accumulation of disability over time [1]. The number of people affected by MS is increasing globally, with recent figures estimating over 2.8 million cases worldwide [2]. While assessments of functional ability can inform medical decisions and interventions that can ultimately reduce the risk of relapses and slow down the rate of disease progression, their utility has been limited by their infrequent use and reliance on patient recall [3-5]. An objective and more frequent assessment could provide a more detailed picture of the evolution of the disease.

To address this unmet need, smartphone sensor–based tests, or remote digital assessment technologies, are increasingly being studied for assessing functional ability in people with MS [6-14]. Typically, such tests can be remotely and frequently performed in the patient’s home environment without supervision by a health care professional [10,12,15,16]. Furthermore, the use of wearable or embedded sensors allows many different aspects of functional ability to be characterized and objectively quantified [12,13,17,18]. Thus, they provide more granular and more detailed information than captured with the single scores of traditional standard clinical assessments such as the Nine-Hole Peg Test, oral Symbol Digit Modalities Test, or the Timed 25-Foot Walk [19-21]. Ultimately, the goal of digital remote assessment tools in MS is to help uncover insidious disease progression [22-24] and allow timely and appropriate medical intervention, which could lead to better treatment outcomes.

While restricting remote digital assessment technologies to a specific device or a single operating system (OS) platform can offer a simpler integration of hardware and software [12,13,25], it may limit their uptake [26]. Bring-your-own-device (BYOD) solutions, by comparison, can help increase access to remote digital technologies (ie, reaching more users) by taking into account the differences in market share of iOS and Android devices across geographies [27]. BYOD solutions are also less affected by the unfamiliarity associated with novel tools, speak to the patients’ preference of using their own device rather than using and carrying an additional device with them, and potentially improve the user experience [26,28,29]. Furthermore, they are associated with fewer logistical challenges and, as a result, are more cost-effective to deploy in clinical trials [30,31]. However, measurement equivalence across different device models must be first established before a BYOD solution can be successfully deployed [11,32].

The Floodlight Open app assesses MS-related functional ability through smartphone sensor–based “active” tests (ie, assessments that require active input from the user) and was specifically developed for use in a BYOD setting [33]. It was deployed in Floodlight Open, a global, open-access, digital-only study that was designed to understand the drivers and barriers in the deployment and persistence of use of a smartphone app in a naturalistic setting and broad study population [33]. Previously, it was shown that the Floodlight Open app can differentiate and discriminate between MS participants and non-MS participants [33,34]. Using data from this study, we sought here to establish the properties of measurement equivalence on 3 separate levels: OS platforms, OS versions, and device models.

## Methods

### Study Design and Participants

Floodlight Open was run in 17 countries, and data were collected between April 23, 2018, and April 26, 2023. The study design and the Floodlight Open app have been previously described [33]. As a fully remote, digital-only study, Floodlight Open did not involve supervision by health care professionals. Adult participants aged 18 years and older with or without self-declared MS living in one of the participating countries could enroll by providing full electronic consent and downloading the Floodlight Open app on their own smartphone device. After providing their electronic consent, participants received a token, or activation code, via email with which they could unlock the functionalities of the app. Participants were excluded from the analyses if they did not complete at least one valid active test or had missing device information.

### Ethical Considerations

The protocol, the electronic informed consent forms, data protection, and relevant supporting information were reviewed and approved by local institutional review boards or ethics committees before the study was initiated, as applicable, in accordance with each country’s regulatory requirements. For example, the institutional review board for the United States was the Western Institutional Review Board in Puyallup, Washington (approval: 20180617). Further details on the institutional review boards and ethics committees’ approvals are described in a previous report [33].

### Floodlight Smartphone Sensor-Based Active Tests

The Floodlight Open app was designed to remotely measure, in a naturalistic setting, functional ability in cognition, hand motor function, gait and balance, mobility, and mood through smartphone-based active tests, passively collected life-space measurements, and patient-reported outcomes. The individual tests have been previously described [33]. It supports all devices running iOS version 11.x or later or Android version 7.x or later, which were commonly available at the time the app was launched. The iOS version was first released on April 22, 2018, and the Android version on July 17, 2019.

The measurement equivalence properties were studied on 6 active tests, including the Information Processing Speed (IPS), Information Processing Speed Digit-Digit (IPS DD), Pinching Test (PT), Static Balance Test (SBT), U-Turn Test (UTT) and 2-Minute Walk Test (2MWT; Table 1). These active tests could be performed up to once daily (PT, SB, UTT, and 2MWT) or up to once weekly (IPS and IPS DD).

**Table 1:** Active tests included in the analysis.

Functional domain and active test		Sensors used	Test schedule	Test feature to assess test performance	Quality control flags	
					Test marked invalid if characterized by	Criterion
**Cognition**						
	IPS^a^	Touchscreen	Weekly	Number of correct responses (n)	“Play-to-quit” attempt	Response selected independently of symbol to be matched [15]
	IPS DD test^b^	Touchscreen	Weekly	Number of correct responses (n)	“Play-to-quit” attempt	Response selected independently of symbol to be matched
**Hand motor function**						
	PT^c^	Touchscreen	Daily	Number of pinches (n)	“Play-to-quit” attempt	No gestures recorded by the touch screen (no screen interaction) [15]
**Gait and balance**						
	**SBT^d^**					
		Accelerometer	Daily	Sway path, (m/s^2^)	“Play-to-quit” attempt	Phone is kept on the table [15] Steps recorded during the test [15]
	**UTT^e^**					
		Accelerometer, Gyroscope	Daily	Turn speed, (rad/s)	“Play-to-quit” attempt	Phone is kept on the table [15] Main orientation of the phone is stable for ≤90% of the time
		Accelerometer, Gyroscope	Daily	Turn speed, (rad/s)	Insufficient data	Number of turns ≤ 3 Turn angle is either ≥270 or ≤90 degrees Test duration is ≤40 s
	**2MWT^f^**					
		Accelerometer	Daily	Number of steps (n)	“Play-to-quit” attempt	Phone is kept on the table [15]
		Accelerometer	Daily	Number of steps (n)	Insufficient data	Test duration ≤105 s

^a^IPS: Information Processing Speed.

^b^IPS DD: Information Processing Speed Digit-Digit.

^c^PT: Pinching Test.

^d^SBT: Static Balance Test.

^e^UTT: U-Turn Test.

^f^2MWT: 2-Minute Walk Test.

The IPS measured cognitive function and instructed participants to match as many symbols to digits as possible within 90 seconds according to a symbol-digit key provided at the top end of the smartphone display. To account for the visuomotor component involved in this substitution task, participants were additionally asked to match digits to digits instead during the 30-second IPS DD. The PT evaluated the ability to perform upper extremity function tasks. The goal was to pinch, or squeeze, as many tomato shapes as possible within 30 seconds. After each pinched tomato shape, a new shape appeared at a different location on the smartphone display. Gait and balance were assessed with 3 different active tests. The SBT instructed participants to stand as still as possible for 30 seconds with both feet on the ground and with their eyes open. The UTT prompted participants to perform at least 5 U-turns on an even ground 4 meters apart within 60 seconds and, thus, assesses both gait and dynamic balance. By comparison, the 2MWT assessed gait during straight walking without turning on an even ground for 2 minutes. For both the UTT and 2MWT, study participants were instructed to walk as fast as possible but safely; use of assistive devices or orthotics was permitted as needed and recorded.

The raw sensor data (touchscreen, accelerometer, and gyroscope data) were encrypted and transferred wirelessly to a secure central database server that is controlled and maintained by the study initiator, F. Hoffmann-La Roche Ltd. Subsequently, the raw sensor data were used to compute predefined test features that characterize the participant’s performance on the individual active tests. These features include the number of correct responses on the IPS and IPS DD, the number of successful pinches on the PT [15], the sway path on the SBT [15], the average turn speed on the UTT [15], and the number of steps on the 2MWT.

### Data Processing and Statistical Analysis

Two data processing steps were implemented to reduce any potential bias introduced by poor sampling frequency of the embedded sensors or by nonaccordant test execution. The second data processing step, however, was not available for the Draw a Shape Test; consequently, this active test is not included in the analyses presented here.

In the first data processing step, any active test recorded with either a low sampling frequency (sampling frequency ≤33 Hz) or an unstable sampling frequency (sampling frequency temporarily dropped ≤33 Hz) was identified and subsequently excluded from the analyses since low or unstable sampling frequency can negatively affect sensor-based measurements [35]. The minimum sampling frequency was set at 33 Hz for all sensor types, as this is the lowest sampling frequency to reliably assess gait [36]. Most currently available commercial smartphone devices support this sampling frequency. Next, any active test that was not executed according to the test’s instructions was disregarded as they were considered invalid. Such nonaccordant tests were retrospectively identified with quality control flags, which were derived from the raw sensor data and provided objective information on how the test was executed. Different quality control flags were defined for each of the active tests (Table 1). These quality control flags identified, without any input from an observer, individual tests that were either characterized by “play-to-quit” behavior or insufficient data. “Play-to-quit” behavior captures instances where the participant did not intend to perform the test according to the instructions provided and wanted to skip the test instead (eg, the same response is selected in fast succession irrespective of the symbol shown during the IPS or IPS DD; no touch screen interaction during the PT). The insufficient data criterion was introduced to ensure the extraction of meaningful gait features, which is particularly important when comparing smaller subgroups that are more sensitive to noise. According to this criterion, only gait tests with a sufficient number of steps or turns were kept for further analysis.

Measurement equivalence signifies that the measurements obtained from 2 groups stem from the same distribution, that is, the 2 groups are equivalent to each other. This was studied separately for each of the 6 active tests in both MS and non-MS participants using the Floodlight feature values, that is, measurements of test performance, derived from the raw sensor data (raw accelerometer, gyroscope, or touchscreen data). Since the study duration was not fixed, the first valid test execution (ie, the first valid IPS, IPS DD, PT, SBT, UTT, and 2MWT) of each participant was used to assess measurement equivalence in order to maximize the number of participants available for analysis and to compare like with like. A test run was considered completed if the participants performed all active tests except the 2MWT in a single session in a predefined, fixed sequence. As the 2MWT was self-administered independently from this sequence, the first valid 2MWT of each participant was used to study the equivalence of this active test.

Feature values derived from the raw sensor data (ie, raw accelerometer, gyroscope, or touchscreen data collected while the participants were performing the active tests) were adjusted for age, sex, and self-declared disease status (“MS” and “Non-MS”) with a robust linear model to account for differences in these covariates (function rlm{} included in the Python *statsmodel* package version 0.13.5). Next, subgroups were defined in 4 separate categories: OS platform (ie, iOS and Android), iOS version (ie, iOS versions 11, 12, 13, 14, and 15), Android version (Android versions 8, 9, and 10), and device model. To minimize potential issues with small sample sizes but also allow for as many different subgroups (eg, device models) as possible to be included in the analysis, only subgroups with at least 20 participants were included.

To study measurement equivalence, each subgroup (eg, iOS 12) was subsequently compared against their respective reference group. This reference group consisted of all remaining subgroups of the same category pooled together. For example, the reference group for iOS 12 is iOS 11, 13, 14, and 15 pooled together. The null hypothesis was that the distribution of the subgroup—for example, iOS 12—is identical to the distribution of its respective reference group. Differences in the means between the subgroup under study and its reference group were tested for statistical significance through permutation testing [37,38]. This test was chosen as it is a nonparametric test that requires only minimal model assumptions and additionally is robust. It does not assume a normal distribution and is also robust with regard to unbalanced data sets such as ours and to outliers. To perform the permutation test, the overall population (in the example above, all iOS devices) was randomly sampled to generate 2 permuted groups whose sample sizes were identical to the subgroup under study (eg, iOS 12) and its reference group (eg, iOS 11, 13, 14, and 15 pooled together), respectively. This was repeated 10,000 times, resulting in 10,000 permutations, and hence 10,000 mean differences. The reported *P* value is defined as the proportion of permutations that resulted in a mean difference between the 2 permuted groups that is larger than the real observed mean difference between the subgroup and its reference group. Both unadjusted and adjusted *P* values (adjusted for multiple comparisons with false discovery rate correction using the Benjamini-Hochberg method) are reported.

Additional reported metrics include the 95% CIs of expected mean differences under the assumption of the null hypothesis, which were derived from the permutation test; the absolute difference and percent difference in the observed mean scores (mean feature values) between each subgroup and their respective reference group; the SD of each subgroup and its reference group; as well as the effect size of these differences (Cohen *d*; very small effect size: *d*=0.01; small effect size: *d*=0.2; medium effect size: *d*=0.5; large effect size: *d*=0.8) [39,40].

To corroborate the findings, a sensitivity analysis was run, which used the median as the test statistic difference (instead of the mean difference) of the permutation test.

In a separate analysis, smartphones were compared against tablets to evaluate the impact of screen size on measurement equivalence. Differences between smartphones and tablets were assessed for statistical significance with the Mann-Whitney *U* Test.

## Results

### Overview

**Figure 1:**
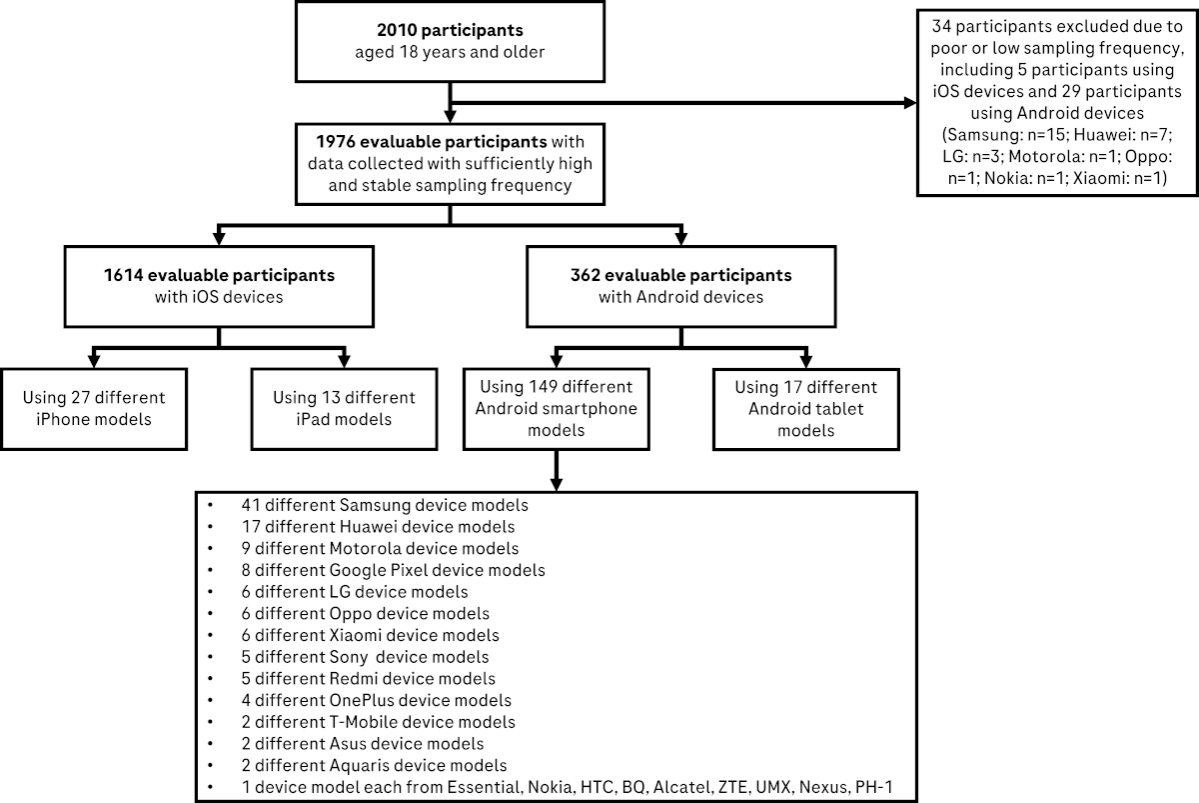
Participant deposition and the devices they used.

Smartphone data were available for 2010 participants aged 18 years and older. Of these, 34 (1.7%) participants were excluded from the analyses due to low or unstable sampling frequency, resulting in 1976 evaluable participants using 206 different, or unique, device models (1614 participants with iOS devices and 362 participants with Android devices). Details on the participant, the devices they used, and their demographics are provided in Figure 1 and Table 2.

**Table 2:** Baseline demographics and disease characteristics.

Variable		All (n=1976)	MS^a^ (n=1140)	Non-MS (n=836)
Age (years), mean (SD)		43.1 (12.5)	45.5 (12.0)	39.8 (12.4)
Female, n (%)		1243 (62.9)	838 (73.5)	405 (48.4)
Participants with iOS devices, n (%)		1614 (81.7)	879 (77.1)	735 (87.9)
**Active tests, mean (SD)**				
	IPS^b^ (correct responses, n)	43.8 (11.9)	43.2 (10.7)	45.0 (13.7)
	IPS DD^c^ (correct responses, n)	17.9 (3.6)	17.7 (3.4)	18.3 (4.0)
	PT^d^ (successful pinches, n)	30.8 (13.5)	28.1 (13.0)	34.6 (13.5)
	SBT^e^ (sway path, m/s^2^)	21.8 (21.9)	22.7 (22.0)	20.5 (21.6)
	UTT^f^ (turn speed, rad/s)	1.40 (0.35)	1.37 (0.34)	1.43 (0.37)
	2MWT^g^ (steps, n)	190.2 (38.2)	191.2 (38.1)	187.8 (38.2)

^a^MS: multiple sclerosis.

^b^IPS: Information Processing Speed.

^c^IPS DD: Information Processing Speed Digit-Digit.

^d^PT: Pinching Test.

^e^SBT: Static Balance Test.

^f^UTT: U-Turn Test.

^g^2MWT: 2-Minute Walk Test.

### Quality Checks

Devices running iOS devices were less prone to low or unstable sampling frequency than Android devices, although the overall number of participants that were excluded for failing to meet the sampling frequency requirements was small. In total, 5 participants with iOS devices and 29 participants with Android devices (15 participants with Samsung; 7 participants with Huawei; 3 participants with LG; and 1 participant each with Motorola, Oppo, Nokia, and Xiaomi devices) were excluded. Of all tests performed during the study, 95.9% (91,002/94,925 tests) were recorded with a sufficiently high and stable sampling frequency.

Across the entire study duration, the proportion of active tests executed in accordance with the test instructions, that is, which passed the criteria defined by the quality control flags, varied from approximately 61.9% to 99.1% (IPS: 6436/6985 [92.1%]; IPS DD: 6278/6609 [95.0%]; PT: 31235/31508 [99.1%]; SBT: 23696/26954 [87.9%]; UTT: 14985/24219 [61.9%]; 2MWT: 18055/21483 [84.0%]). However, no participant was excluded from the analysis for failing to meet these criteria as each participant performed at least one entire test run in accordance with the provided instructions (for each participant, the first valid test from each active test was used for the measurement equivalence analysis). Nonetheless, this highlights that large observational remote assessment studies such as Floodlight Open require checks that provide objective measures for assessing data quality in accordance with the test instructions of self-administered assessments [15].

### Measurement Equivalence

The measurement equivalence analysis was assessed after adjusting for age, sex, and self-reported disease status. Overall, results show no indication that any of the 6 active tests are associated with a systematic measurement nonequivalence. The findings were consistent across OS platforms, OS versions, and device models, although the findings for the device models were more variable given the smaller subgroups. The effect sizes of the difference between subgroups were mostly very small (effect size <0.20) or small (effect size <0.5). For each observed subgroup, the mean differences were considerably smaller than one SD of the respective reference group, and most mean differences were within the 95% CI obtained from the permutation test (Tables 3-5; Table S1 in Multimedia Appendix 1).

**Figure 2:**
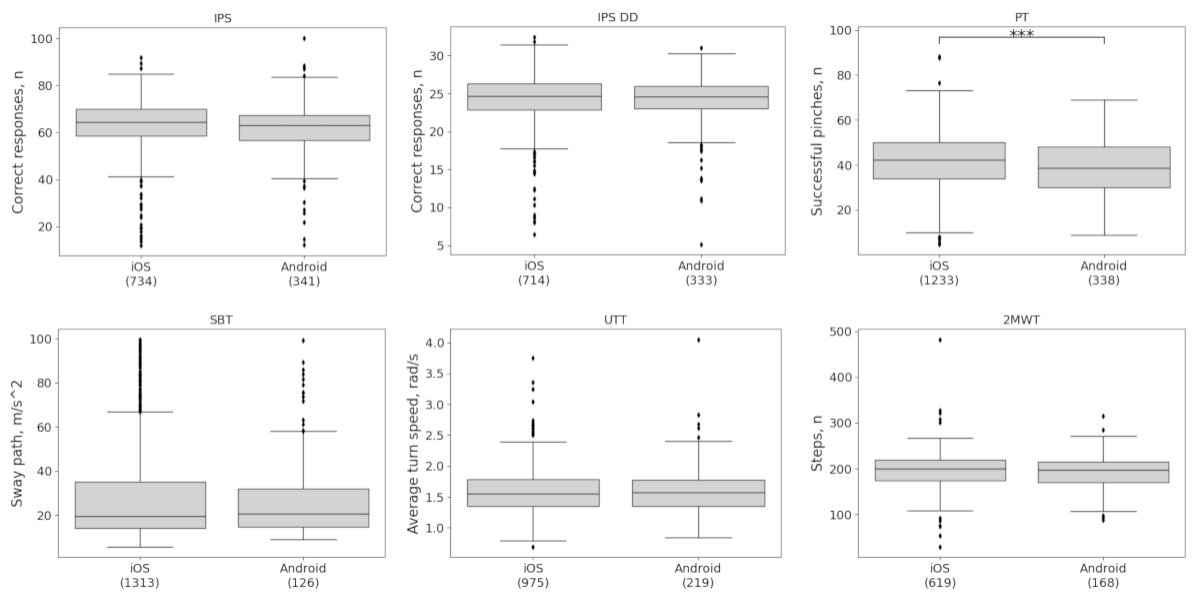
Measurement equivalence by operating system (OS) platform. Permutation testing showed no evidence of a systematic lack of equivalence between the 2 OS platforms—iOS and Android. A small but statistically significant difference was only observed on the PT. Differences between groups as well as effect sizes are provided in Table 3. Brackets indicate the sample size. ***P<.001. IPS: Information Processing Speed; IPS DD: Information Processing Speed Digit-Digit; PT: Pinching Test; SBT: Static Balance Test; UTT: U-Turn Test; 2MWT: 2-Minute Walk Test.

When comparing iOS devices with Android devices, percent differences in adjusted test scores between iOS devices and Android devices were <5% on the IPS, IPS DD, SBT, UTT, and 2MWT and ≤10% on the PT (Table 3). Permutation testing revealed a statistically significant, but small difference between iOS and Android on the PT (percent difference: 8.0%, effect size: 0.24, *P*<.001; Figure 2; Table 3).

**Figure 3:**
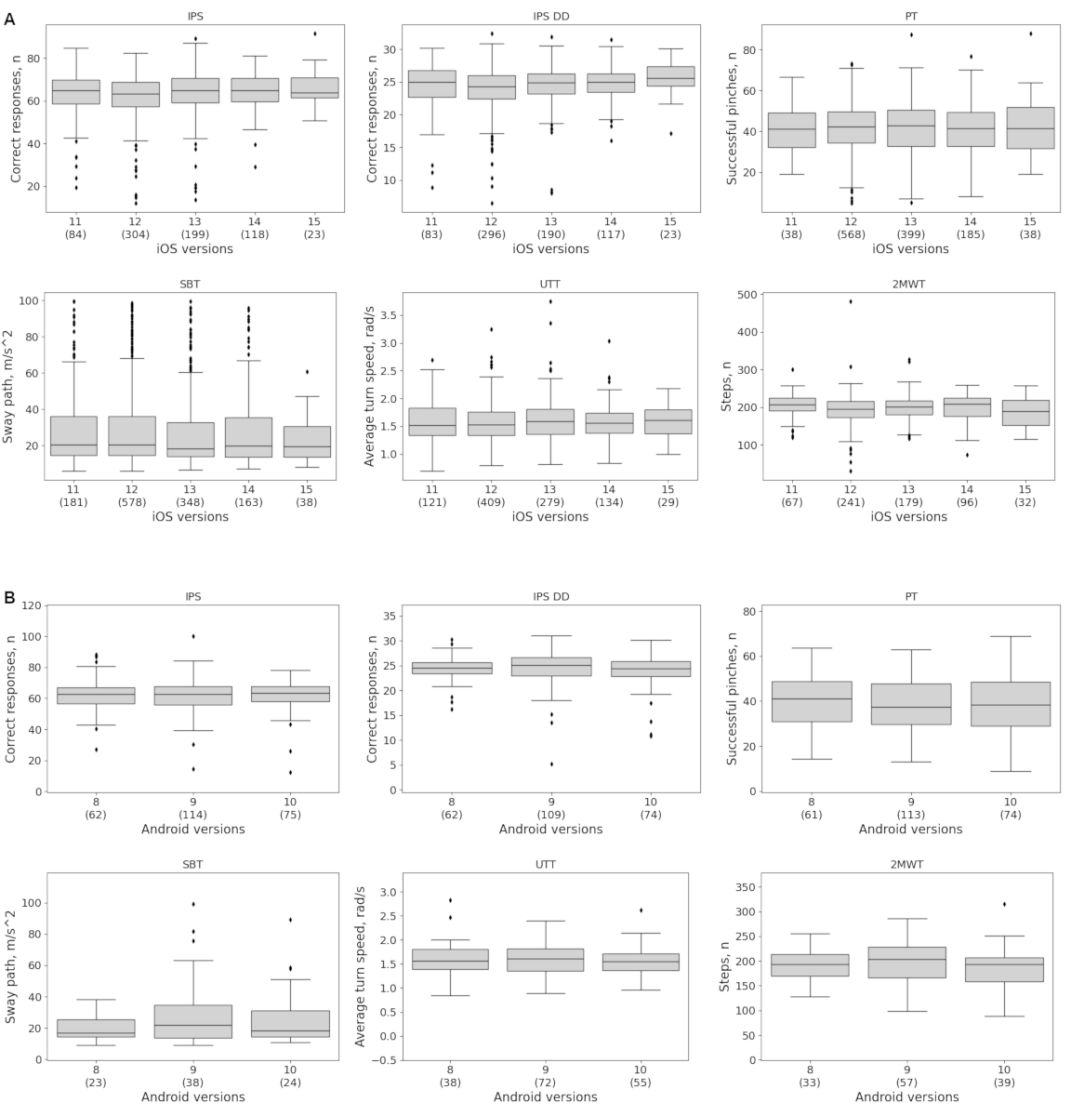
Measurement equivalence by (A) iOS version and (B) Android version. Permutation testing revealed no evidence of a systematic lack of equivalence. No statistically significant differences were observed across operating system versions after correcting for multiple comparisons (all P_adjusted_>.05). Absolute and percent differences as well as effect sizes are provided in Tables 4 and 5. Brackets indicate the sample size. IPS: Information Processing Speed; IPS DD: Information Processing Speed Digit-Digit; PT: Pinching Test; SBT: Static Balance Test; UTT: U-Turn Test; 2MWT: 2-Minute Walk Test.

Similar results were observed when comparing the 5 iOS versions 11, 12, 13, 14, and 15 (Figure 3A). Across all 5 iOS versions, the percent differences were mostly <5% on the IPS and IPS DD, PT, UTT, and 2MWT, with only iOS 15 showing percent differences greater than 5% on the IPS (percent difference: 5.3%) and IPS DD (percent difference: 5.2%) (Table 4). Larger differences were observed on the SBT, with percent differences ranging from 1.3% to 18.3%. Permutation testing revealed statistically significant differences only for iO12 on the IPS (percent difference: 3.0%, effect size: 0.17, *P*_unadjusted_=.02) and IPS DD (percent difference: 2.5%, effect size: 0.19, *P*_unadjusted_=.01). However, the observed mean differences of 1.9 (IPS) and 0.6 (IPS DD) correct responses were within the 95% CI obtained from the permutation test (IPS: [0.0,1.9]; IPS DD: [0.0,0.6]), and these differences were no longer statistically significant after correcting for multiple comparisons (*P*_adjusted_=.11 and .07, respectively). Differences of similar magnitude were observed when comparing Android versions 8, 9, and 10 (Figure 3B). Across these OS versions, percent differences were mostly ≤5% on the IPS, IPS DD, PT (only Android 8 showed a larger percent difference of 5.1%), UTT, and 2MWT; and <30% on the SBT (Table 5). None of these differences reached statistical significance (all *P*_unadjusted_=.06-.96, all *P*_adjusted_=.19-.96).

Next, we evaluated whether a device model shows an out-of-distribution performance compared with the null hypothesis of all device models being from the same distribution (Table S1 and Figure S1 in Multimedia Appendix 1). Only device models used by at least 20 participants were included in this analysis, resulting in 17 different models being evaluated. Percent differences were mostly ≤5% on the IPS, IPS DD, UTT (only the iPhone 7 Plus showed a larger percent difference of 5.6%) and 2MWT; and <20% on the PT and SBT (Table S1 in Multimedia Appendix 1). Unadjusted permutation testing revealed that most device models did not show a statistically significant difference (IPS: 12/13 models, IPS DD: all models, PT: 12/16 models, SBT: 12/14 models, UTT: all models, 2MWT: all models). Of note, the statistically significant differences (*P*_unadjusted_<.001-.04) were not associated with any particular device model. After adjusting for multiple comparisons, statistically significant differences were observed only on the PT (iPhone SE: *P*_adjusted_=.02; iPhone X Global: *P*_adjusted_=.03; iPhone X GSM: *P*_adjusted_≤.001; iPhone 11: *P*_adjusted_=.03). In all 4 instances, effect sizes ranged from 0.38 to 0.50, and percent differences from 11.4% to 15.1%. Furthermore, the observed mean differences were outside the 95% CI obtained from the permutation test (Table S1 in Multimedia Appendix 1).

The sensitivity analysis, which used the median difference rather than the mean difference as the test statistic for the permutation test, revealed similar findings across OS platforms, OS versions, and device models (Tables S2-S5 in Multimedia Appendix 1).

Screen size had a limited impact on the measurement equivalence properties. No significant differences were observed between smartphones (n=1788, of which 1556 [87.0%] were iOS devices) and tablets (n=89, of which 58 [65.2%] were iPads running iOS) on the gait and balance tests (ie, SBT, UTT and 2MWT, all *P*=.27-.75). Statistically significant differences with small effect sizes between smartphones and tablets were observed on the IPS (percent difference: 11.9%, effect size: 0.45, *P*=.001), IPS DD (percent difference: 7.3%, effect size: 0.37, *P*=.022), and PT (percent difference: 18.1%, effect size: 0.38, *P*=.002).

**Table 3:** Absolute and percent mean differences across operating system platforms (iOS vs Android).

Active test	iOS			Android			Mean difference from Android devices				Permutation test	
	n	Mean (SD)	n		Mean (SD)	Absolute difference		Percent difference	Effect size	95% CI		*P*_unadjusted_
IPS^a^ (correct responses, n)	734	63.1 (11.2)	341		61.8 (10.3)	1.3		2.1	0.12	0.0-1.6		.07
IPS DD^b^ (correct responses, n)	714	24.3 (3.4)	333		24.2 (3.1)	0.1		0.5	0.04	0.0-0.5		.60
PT^c^ (successful pinches, n)	1233	41.5 (12.7)	338		38.4 (12.1)	3.1		8.0	0.24	0.0-1.7		<.001
SBT^d^ (sway path, m/s^2^)	1313	28.2 (20.7)	126		28.4 (20.8)	0.2		0.7	0.01	0.1-4.3		.92
UTT^e^ (turn speed, rad/s)	975	1.59 (0.34)	219		1.59 (0.37)	0.01		0.4	0.02	0.00-0.06		.80
2MWT^f^ (steps, n)	619	195.9 (38.0)	168		191.9 (38.2)	4.0		2.1	0.11	0.1-7.5		.23

^a^IPS: Information Processing Speed.

^b^IPS DD: Information Processing Speed Digit-Digit.

^c^PT: Pinching Test.

^d^SBT: Static Balance Test.

^e^UTT: U-Turn Test.

^f^2MWT: 2-Minute Walk Test.

**Table 4:** Absolute and percent differences across iOS versions.

Active test		iOS subgroup			Reference group^a^, mean (SD)		Mean difference from reference group						Permutation test			
		n	Mean (SD)				Absolute difference		Percent difference	Effect size		95% CI			*P*_unadjusted_	*P*_adjusted_^b^
**IPS^c^ (correct responses, n)**																
	iOS 11	84	62.9 (12.8)	63.1 (11.0)		0.2		0.4		0.02	0.0-2.9			.86		.87
	iOS 12	304	62.0 (11.5)	63.9 (11.0)		1.9		3.0		0.17	0.0-1.9			.02		.11
	iOS 13	199	63.7 (11.8)	62.9 (11.0)		0.8		1.2		0.07	0.0-2.1			.40		.50
	iOS 14	118	64.5 (8.5)	62.8 (11.7)		1.7		2.7		0.15	0.0-2.5			.12		.27
	iOS 15	23	66.3 (9.0)	63.0 (11.3)		3.3		5.3		0.30	0.1-5.3			.16		.27
**IPS DD^d^ (correct responses, n)**																
	iOS 11	83	24.2 (3.9)	24.3 (3.3)		0.1		0.5		0.03	0.0-0.9			.78		.78
	iOS 12	296	23.9 (3.5)	24.5 (3.3)		0.6		2.5		0.19	0.0-0.6			.01		.07
	iOS 13	190	24.5 (3.3)	24.2 (3.4)		0.2		0.9		0.07	0.0-0.6			.43		.53
	iOS 14	117	24.8 (2.7)	24.2 (3.5)		0.6		2.4		0.17	0.0-0.8			.09		.16
	iOS 15	23	25.5 (3.1)	24.2 (3.4)		1.3		5.2		0.38	0.0-1.6			.08		.16
**PT^e^ (successful pinches, n)**																
	iOS 11	38	41.2 (11.7)	41.5 (12.7)		0.3		0.7		0.02	0.1-4.7			.89		.99
	iOS 12	568	41.6 (12.0)	41.3 (13.3)		0.3		0.7		0.02	0.0-1.6			.71		.99
	iOS 13	399	41.5 (13.6)	41.5 (12.3)		0.0		0.0		0.00	0.0-1.8			.99		.99
	iOS 14	185	40.9 (12.8)	41.6 (12.7)		0.7		1.7		0.06	0.0-2.3			.48		.99
	iOS 15	38	42.6 (15.0)	41.4 (12.6)		1.2		2.9		0.09	0.1-4.8			.58		.99
**SBT^f^ (sway path, m/s^2^)**																
	iOS 11	181	29.1 (21.5)	28.1 (20.5)		1.0		3.7		0.05	0.0-3.7			.54		.67
	iOS 12	578	29.1 (21.0)	27.5 (20.4)		1.6		5.7		0.08	0.0-2.6			.17		.29
	iOS 13	348	26.7 (20.0)	28.7 (20.9)		2.0		7.1		0.10	0.0-2.9			.11		.29
	iOS 14	163	28.5 (21.7)	28.2 (20.5)		0.4		1.3		0.02	0.1-3.9			.83		.83
	iOS 15	38	23.2 (12.0)	28.3 (20.9)		5.2		18.3		0.25	0.1-7.6			.13		.29
**UTT^g^ (turn speed, rad/s)**																
	iOS 11	121	1.58 (0.35)	1.59 (0.34)		0.01		0.4		0.02	0.00-0.08			.85		1.0
	iOS 12	409	1.57 (0.33)	1.60 (0.35)		0.03		2.0		0.09	0.00-0.05			.15		.38
	iOS 13	279	1.62 (0.38)	1.58 (0.33)		0.04		2.7		0.12	0.00-0.06			.08		.38
	iOS 14	134	1.59 (0.31)	1.59 (0.35)		0.00		0.0		0.00	0.00-0.07			1.0		1.0
	iOS 15	29	1.59 (0.31)	1.59 (0.35)		0.00		0.2		0.01	0.00-0.15			.96		1.0
**2MWT^h^ (steps, n)**																
	iOS 11	67	204.1 (33.7)	194.8 (38.4)		9.3		4.8		0.25	0.2-10.9			.057		.16
	iOS 12	241	192.2 (42.1)	198.1 (35.0)		5.9		3.0		0.15	0.1-7.1			.06		.16
	iOS 13	179	197.7 (34.1)	195.0 (39.5)		2.6		1.4		0.07	0.1-7.6			.43		.45
	iOS 14	96	198.4 (36.1)	195.3 (38.4)		3.1		1.6		0.08	0.1-9.5			.45		.45
	iOS 15	32	186.9 (38.7)	196.3 (38.0		9.4		4.8		0.25	0.2-15.7			.17		.29

^a^The reference group consists of all other iOS devices pooled together.

^b^*P* values were adjusted for multiple comparisons with false discovery rate (FDR) correction using the Benjamini-Hochberg method.

^c^IPS: Information Processing Speed.

^d^IPS DD: Information Processing Speed Digit-Digit.

^e^PT: Pinching Test.

^f^SBT: Static Balance Test.

^g^UTT: U-Turn Test.

^h^2MWT: 2-Minute Walk Test.

**Table 5:** Absolute and percent differences across Android versions.

Active test		Android subgroup		Reference group^a^, mean (SD)	Mean difference from reference group				Permutation Test		
		n	Mean (SD)		Absolute difference	Percent difference	Effect size	95% CI		*P*_unadjusted_	*P*_adjusted_^b^
**IPS^c^ (correct responses, n)**											
	Android 8	62	62.4 (11.5)	61.8 (10.5)	0.6	0.9	0.05	0.1-3.6		.71	.90
	Android 9	114	61.8 (10.5)	62.1 (10.9)	0.3	0.5	0.03	0.0-3.0		.83	.90
	Android 10	75	61.9 (10.6)	62.0 (10.8)	0.2	0.3	0.02	0.0-3.3		.90	.90
**IPS DD^d^ (correct responses, n)**											
	Android 8	62	24.4 (2.5)	24.2 (3.5)	0.3	1.1	0.09	0.0-1.1		.57	.85
	Android 9	109	24.3 (3.6)	24.2 (3.0)	0.1	0.3	0.03	0.0-0.9		.85	.85
	Android 10	74	24.0 (3.4)	24.3 (3.2)	0.3	1.4	0.11	0.0-1.0		.46	.85
**PT^e^ (successful pinches, n)**											
	Android 8	61	40.0 (12.0)	38.1 (12.0)	1.9	5.1	0.16	0.1-3.9		.27	.57
	Android 9	113	37.8 (11.3)	39.2 (12.6)	1.3	3.4	0.11	0.1-3.5		.38	.57
	Android 10	74	38.5 (13.3)	38.6 (11.5)	0.1	0.3	0.01	0.1-3.7		.94	.94
**SBT^f^ (sway path, m/s^2^)**											
	Android 8	23	19.8 (7.8)	28.2 (20.6)	8.4	29.8	0.46	0.2-10.0		.06	.19
	Android 9	38	28.7 (21.7)	23.8 (15.3)	4.9	20.6	0.27	0.1-9.1		.23	.35
	Android 10	24	27.5 (19.8)	25.3 (18.0)	2.2	8.7	0.12	0.1-9.9		.63	.64
**UTT^g^ (turn speed, rad/s)**											
	Android 8	38	1.59 (0.37)	1.59 (0.32)	0.00	0.2	0.01	0.00-0.14		.96	.96
	Android 9	72	1.62 (0.34)	1.57 (0.32)	0.05	3.1	0.15	0.00-0.12		.36	.54
	Android 10	55	1.55 (0.30)	1.60 (0.35)	0.05	3.2	0.15	0.00-0.12		.35	.54
**2MWT^h^ (steps, n)**											
	Android 8	33	191.1 (33.8)	191.8 (42.6)	0.7	0.4	0.02	0.3-18.3		.94	.94
	Android 9	57	195.7 (41.0)	188.3 (40.0)	7.3	3.9	0.18	0.2-16.1		.31	.46
	Android 10	39	186.0 (45.3)	194.0 (38.1)	7.9	4.1	0.20	0.3-17.3		.30	.46

^a^The reference group consists of all other Android devices pooled together.

^b^*P* values were adjusted for multiple comparisons with false discovery rate (FDR) correction using the Benjamini-Hochberg method.

^c^IPS: Information Processing Speed.

^d^IPS DD: Information Processing Speed Digit-Digit.

^e^PT: Pinching Test.

^f^SBT: Static Balance Test.

^g^UTT: U-Turn Test.

^h^2MWT: 2-Minute Walk Test.

## Discussion

### Principal Findings

Ensuring measurement equivalence across different device models is necessary to conduct a new form of large-scale, global, and cost-effective clinical research on digital outcomes in a BYOD setting. In this analysis, no evidence was found for a systematic lack of measurement equivalence across devices for the 6 smartphone sensor–based active tests considered. The key learnings are summarized in the Textbox 1.

Measurement nonequivalence can in theory arise from various sources. For example, differences in inertial measurement unit (IMU) sensors can potentially contribute to differences in measurements obtained between devices [35]. However, our results suggest that differences in IMU sensors did not impact the features derived from our active tests as no statistically significant differences were observed on tests that rely on IMU sensors (ie, the SBT, UTT, and 2MWT).

**Key learnings.**

- The broad, multinational cohort of 1976 participants and 206 different device models allowed us to study measurement equivalence in a naturalistic environment, reflective of a real-world setting.
- No evidence for the systematic lack of measurement equivalence was found; the features derived from our smartphone-based assessments were by large robust against differences in inertial measurement unit (IMU) sensors, sampling frequency, and device latency.
- Designing features to be robust against cumulative device latency effects can help ensure measurement equivalence across operating system (OS) platforms, OS versions, and device models.
- Objective criteria that assess whether a self-administered digital test was taken as instructed and recorded with a sufficiently high and stable sampling frequency can help with evaluating the quality of the data collected in an unsupervised setting and, hence, informing whether the data should be included in further analyses.

Another factor that could contribute to measurement nonequivalence is differences in sampling frequency or sampling frequency heterogeneity [35]. We, therefore, excluded any tests that were recorded with either too low a sampling frequency or with an unstable sampling frequency as the sampling frequency can vary from test execution to execution, even with the same device model. Nonetheless, the sampling frequency requirements were met in most cases even if the computational load was temporarily increased due to the simultaneous activity of other apps installed on the participants’ own smartphone devices as can be expected in a BYOD setting. Of the 2010 participants considered for the analyses, only 34 participants were excluded from the analysis for the lack of a sufficiently high and stable sample frequency.

A third factor that could impact measurement equivalence is device latency. For touchscreen-based tests, it was estimated that latencies associated with displaying a new visual cue on the screen and registering a touchscreen input by the user could account for a variance in response time of up to 100 ms, with Android devices tending to show higher latencies than iOS devices [41]. We estimated that such latencies could, in the worst case, account for a difference of up to 3 pinches on the PT (100 ms latency per pinched tomato × an average 30.8 pinched tomatoes during a PT [Table 2] = 3.08 seconds latency during a PT; with approximately 1 pinched tomato per second, this cumulative latency may thus explain a difference of up to 3 successful pinches). This is comparable to the 3.1 fewer successful pinches observed on Android devices than on iOS devices. While other factors cannot be ruled out, it is plausible that the difference of 3.1 successful pinches between Android and iOS devices observed in this study could be explained by differences in device latencies. Developing features that are not affected by such cumulative latency effects could improve the measurement equivalence between iOS and Android devices. For the PT, a possible feature is a double touch asynchrony, which was developed for other studies that deployed the Floodlight technology as a measure of finger coordination. It measures the duration of the gap between the first and second fingers touching the touch screen. In a previous analysis, it was reliable, correlated with clinician-administered measures of upper extremity impairment and MS-related disability, and differentiated between people with different levels of disability [42]. Unlike the number of successful pinches, this feature is by design not affected by cumulative latency effects as it measures the average time between the first and second finger touching the touch screen during a single PT.

Our study also highlighted the need for objective measures of data quality and accordance with the test instructions. Across the 6 active tests considered, approximately 61.9% through 99.1% of all test attempts were considered valid. A higher proportion of tests passing the quality-check flags have been previously reported when using less stringent criteria [33]. Ensuring that the tests are executed in accordance with their instructions remains a challenge with fully remote digital health studies which do not involve direct oversight of test performance and feedback mechanisms with the participants. In-clinic visits, for example, could be used to onboard study participants in person and explain the tests in more detail, consequently increasing the proportion of active tests executed in accordance with the test instructions. In fact, we previously reported for a separate study, which included regular clinic visits, that up to 99% of active test attempts were considered valid [15]. Furthermore, digital health studies that include clinic visits, such as [15], are more likely than fully remote studies to enroll participants who are more motivated. This becomes more noticeable the longer the test duration is and the smaller the subgroups that are compared with each other. As a result, we implemented in this study additional quality control flags to ensure sufficient evaluable data were collected during the 2 active tests with the longest test duration, the UTT and 2MWT (Table 1).

### Limitations

There are some limitations associated with our study. First, the observational nature of our study in a naturalistic environment implies that confounding factors may have affected the comparisons despite the adjustments we performed. For instance, we could not adjust for disease severity, as this information is not available in this fully remote, digital-only study. This might partially explain the larger percent differences observed in smaller subgroups. However, given the small overall differences among the investigated subgroups, the residual confounding effects are plausibly small. While statistical tests did not indicate that the measurements obtained across devices are systematically nonequivalent, we cannot rule out that this finding may be due to high variability, different average disease severity, or, for a few comparisons, a small sample size. A lack of sensitivity of the Floodlight tests, however, can be excluded as a reason for the similarity of the results as these tests have shown the ability to differentiate people with MS from healthy controls or between people with MS with different levels of impairment [15,42,43].

Second, the earlier release of the iOS version of the Floodlight Open app has resulted in a larger number of iOS participants compared with Android participants. Furthermore, the larger choice of different Android device models than iOS device models meant that there were fewer participants per Android device model, resulting in an unbalanced data set for the comparison of measurement equivalence by device model.

Finally, the analyses presented here are retrospective as the study was not purposely designed to assess equivalence or reliability. Ideally, device equivalence studies with purpose-built study designs have each participant perform the active tests multiple times using the same set of devices and assess equivalence across devices using appropriate statistical tests with prespecified equivalence margins. As an alternative, equivalence can be assessed with bench testing using robots able to cover the range of feature values typically observed in the population of interest. Nonetheless, the preliminary findings of our analyses can inform the design of future device equivalence studies and the equivalence margins to assess equivalence.

### Comparison With Prior Work

Recent studies compared smartphone-based measurements of gait, balance, or physical activity [44-48], cognitive functioning [49,50], or sleep [51]. However, these studies typically compared the smartphone-based measurements and measurements obtained with other devices with gold-standard measures such as manual step count [46,47] or smartphone-based measurements with measurements obtained with other device types [44,45,51]. This makes it challenging to separate the potential impact arising from differences in the device or sensor specifications from those arising from differences in the algorithms used to extract the features. A small number of studies assessed device equivalence using the same feature extraction algorithm [35,49]. For example, van Oirschot et al [49] investigated their smartphone-based Symbol Digit Modalities Test on devices running either iOS or Android and found no differences between the 2 OS platforms, which is in line with our findings on the IPS. Additionally, Ena et al [52] identified a number of features extracted from their smartphone-based mobility tests that showed in a prospective study reliability across iOS and Android devices.

Strengths of this remote, digital-only, unsupervised, BYOD study include the comprehensive approach to assess measurement equivalence across different smartphone devices. Unlike other studies, we investigated measurement equivalence on 3 levels by separately looking at the 2 OS platforms iOS and Android, different iOS and Android versions, and device models. The large sample size of 1976 study participants and the wide range of smartphone device models (n=206) used add to the strength of our study. Finally, we used objective measures to ensure data quality and accordance with test instructions.

### Conclusions

In this study, we investigated a key criterion for the deployment of 6 smartphone sensor–based active tests assessing cognition, hand motor function, and gait and balance in a BYOD setting. Using data collected in Floodlight Open, we found no evidence for a substantial and consistent lack of measurement equivalence across different OS platforms, OS versions, and smartphone device models for features derived from these active tests. These features were largely unaffected by differences in IMU and touchscreen sensors and were also not impacted by the activity of other apps installed on the participants’ own smartphone devices, as evidenced by the sufficiently high and stable sampling frequency in a vast majority of participants. These findings are compatible with the use of smartphone-based tests in a BYOD setting and will inform future studies with purpose-built study designs that will further investigate the device equivalence properties of smartphone-based assessments.

## Data Availability

Up-to-date details on Roche’s Global Policy on Sharing of Clinical Study Information and how to request access to related clinical study documents are available in [53]. Request for the data underlying this publication requires a detailed, hypothesis-driven, statistical analysis plan that is collaboratively developed by the requestor and company subject matter experts. Such requests should be directed to dbm.datarequest@roche.com for consideration. Anonymized records for individual patients across more than one data source external to Roche cannot, and should not, be linked due to a potential increase in risk of patient reidentification.

## Appendix

Multimedia Appendix 1 Absolute and percent mean difference by device model and sensitivity analysis results.

## Abbreviations

2MWT: 2-Minute Walk Test
BYOD: bring-your-own-device
IMU: inertial measurement unit
IPS DD: Information Processing Speed Digit-Digit
IPS: Information Processing Speed
MS: multiple sclerosis
OS: operating system
PT: Pinching Test
SBT: Static Balance Test
UTT: U-Turn Test
