# Supplementary material for: Investigating Measurement Equivalence of Smartphone Sensor–Based Assessments: Remote, Digital, Bring-Your-Own-Device Study
Source: J Med Internet Res. 2025 Apr 3;27:e63090. doi: 10.2196/63090 (PMC12006779; doi:10.2196/63090)
Supplement: Multimedia Appendix 1 [file jmir_v27i1e63090_app1.docx]

# Supplementary appendix to:

# Investigating measurement equivalence of smartphone sensor-based assessments: A remote, digital, bring-your-own-device study

Lito Kriara, PhD^1^ Frank Dondelinger, PhD^1^ Luca Capezzuto, PhD^1^ Corrado Bernasconi, MD, PhD^1^ Florian Lipsmeier, PhD^1^ Adriano Galati, PhD^1^ Michael Lindemann, PhD^1^

^1^F. Hoffmann-La Roche Ltd., Basel, Switzerland.

**Correspondence:**

Lito Kriara

F. Hoffmann-La Roche Ltd., Grenzacherstrasse 124, CH-4070 Basel, Switzerland

lito.kriara@roche.com

+41 61 687 10 20

**Table S1. Absolute and percent mean difference by device model.**

| **Active test** | **Device subgroup** | | | **Reference group^a^** | | **Mean difference from reference group** | | | **Permutation test** | | |
| --- | --- | --- | --- | --- | --- | --- | --- | --- | --- | --- | --- |
|  | **n** | **Mean** | **SD** | **Mean** | **SD** | **Absolute difference** | **Percent difference** | **Effect size** | **95% CI** | ***P*_unadjusted_** | ***P*_adjusted_^b^** |
| **IPS, correct responses, n** |  |  |  |  |  |  |  |  |  |  |  |
| iPhone 6 | 45 | 64.7 | 7.8 | 62.8 | 11.8 | 1.9 | 3.1 | 0.17 | 0.1 - 5.8 | .46 | .88 |
| iPhone 6s | 70 | 60.9 | 13.8 | 63.2 | 11.2 | 2.3 | 3.6 | 0.20 | 0.1 - 5.8 | .39 | .88 |
| iPhone SE | 36 | 62.5 | 10.5 | 63.0 | 11.6 | 0.4 | 0.7 | 0.04 | 0.1 - 4.3 | .83 | .97 |
| iPhone 7 | 98 | 63.3 | 11.2 | 62.9 | 11.6 | 0.4 | 0.6 | 0.03 | 0.1 - 4.0 | .82 | .97 |
| iPhone 7 Plus | 33 | 65.3 | 8.1 | 62.8 | 11.7 | 2.5 | 4.0 | 0.22 | 0.0 - 3.3 | .08 | .36 |
| iPhone 8 | 108 | 62.9 | 10.9 | 63.0 | 11.7 | 0.1 | 0.1 | 0.00 | 0.0 - 2.9 | .97 | .97 |
| iPhone 8 Plus | 62 | 62.0 | 14.7 | 63.0 | 11.2 | 1.1 | 1.7 | 0.09 | 0.1 - 4.7 | .61 | .88 |
| iPhone X GSM | 31 | 60.4 | 16.9 | 63.1 | 11.2 | 2.7 | 4.3 | 0.23 | 0.0 - 2.7 | .03 | .36 |
| iPhone XS | 20 | 60.2 | 7.7 | 63.0 | 11.6 | 2.8 | 4.5 | 0.24 | 0.0 - 3.5 | .07 | .36 |
| iPhone XR | 68 | 64.2 | 11.5 | 62.8 | 11.5 | 1.4 | 2.2 | 0.12 | 0.1 - 4.4 | .48 | .88 |
| iPhone 11 | 38 | 64.2 | 8.7 | 62.9 | 11.7 | 1.3 | 2.1 | 0.11 | 0.1 - 4.8 | .54 | .88 |
| Samsung Galaxy S7 | 21 | 64.6 | 11.8 | 62.9 | 11.5 | 1.7 | 2.7 | 0.15 | 0.0 - 3.3 | .24 | .79 |
| Samsung Galaxy S9 | 20 | 62.7 | 7.4 | 63.0 | 11.6 | 0.3 | 0.5 | 0.03 | 0.1 - 5.7 | .91 | .97 |
| **IPS DD, correct responses, n** |  |  |  |  |  |  |  |  |  |  |  |
| iPhone 6 | 44 | 24.7 | 2.4 | 24.2 | 3.4 | 0.5 | 2.2 | 0.15 | 0.0 - 1.7 | .48 | .88 |
| iPhone 6s | 68 | 23.8 | 3.9 | 24.3 | 3.3 | 0.4 | 1.8 | 0.13 | 0.0 - 1.3 | .43 | .88 |
| iPhone SE | 35 | 23.6 | 2.9 | 24.3 | 3.4 | 0.7 | 2.7 | 0.20 | 0.0 - 1.2 | .21 | .83 |
| iPhone 7 | 96 | 24.4 | 2.8 | 24.2 | 3.5 | 0.2 | 0.8 | 0.06 | 0.0 - 1.0 | .67 | .88 |
| iPhone 7 Plus | 34 | 24.9 | 2.4 | 24.2 | 3.4 | 0.7 | 2.7 | 0.20 | 0.0 - 0.8 | .07 | .48 |
| iPhone 8 | 101 | 24.3 | 3.7 | 24.2 | 3.3 | 0.1 | 0.3 | 0.02 | 0.0 - 1.3 | .92 | .92 |
| iPhone 8 Plus | 59 | 23.7 | 4.5 | 24.3 | 3.2 | 0.7 | 2.7 | 0.19 | 0.0 - 0.8 | .08 | .48 |
| iPhone X GSM | 31 | 24.0 | 5.1 | 24.3 | 3.3 | 0.3 | 1.1 | 0.08 | 0.0 - 1.0 | .57 | .88 |
| iPhone XS | 20 | 24.0 | 2.9 | 24.2 | 3.4 | 0.2 | 0.9 | 0.06 | 0.0 - 1.3 | .73 | .88 |
| iPhone XR | 65 | 24.5 | 2.9 | 24.2 | 3.4 | 0.3 | 1.2 | 0.08 | 0.0 - 1.4 | .65 | .88 |
| iPhone 11 | 39 | 24.6 | 2.8 | 24.2 | 3.4 | 0.4 | 1.7 | 0.12 | 0.0 - 1.0 | .36 | .88 |
| Samsung Galaxy S7 | 21 | 24.3 | 2.0 | 24.2 | 3.4 | 0.1 | 0.3 | 0.02 | 0.0 - 1.7 | .92 | .92 |
| **PT, successful pinches, n** |  |  |  |  |  |  |  |  |  |  |  |
| iPhone 6 | 55 | 36.8 | 11.2 | 41.9 | 12.6 | 5.1 | 12.2 | 0.41 | 0.1 - 6.4 | .07 | .18 |
| iPhone 6s | 95 | 42.4 | 12.0 | 41.6 | 12.7 | 0.8 | 2.0 | 0.07 | 0.1 - 6.2 | .77 | .93 |
| **iPhone SE** | **46** | **37.1** | **10.9** | **41.8** | **12.6** | **4.8** | **11.4** | **0.38** | **0.0** - **3.5** | **.002** | **.02** |
| iPhone 7 | 156 | 43.8 | 11.3 | 41.3 | 12.8 | 2.5 | 6.0 | 0.20 | 0.1 - 5.6 | .30 | .48 |
| iPhone 7 Plus | 47 | 41.4 | 12.5 | 41.6 | 12.6 | 0.2 | 0.5 | 0.02 | 0.1 - 3.9 | .91 | .97 |
| iPhone 8 | 211 | 42.7 | 11.9 | 41.4 | 12.7 | 1.3 | 3.2 | 0.10 | 0.0 - 3.0 | .33 | .48 |
| iPhone 8 Plus | 85 | 42.8 | 13.0 | 41.5 | 12.6 | 1.3 | 3.1 | 0.10 | 0.0 - 2.5 | .24 | .43 |
| **iPhone X Global** | **32** | **46.5** | **11.3** | **41.5** | **12.6** | **5.0** | **12.1** | **0.40** | **0.1** - **4.2** | **.008** | **.03** |
| **iPhone X GSM** | **53** | **46.3** | **13.7** | **41.4** | **12.5** | **4.9** | **11.9** | **0.39** | **0.0** - **2.1** | **<.001** | **<.001** |
| iPhone XS | 51 | 44.2 | 13.5 | 41.5 | 12.5 | 2.7 | 6.4 | 0.21 | 0.0 - 3.2 | .06 | .18 |
| iPhone XS Max Global | 32 | 41.6 | 13.8 | 41.6 | 12.6 | 0.1 | 0.2 | 0.01 | 0.1 - 4.2 | .97 | .97 |
| iPhone XR | 132 | 39.2 | 13.5 | 42.0 | 12.5 | 2.7 | 6.5 | 0.22 | 0.1 - 4.0 | .13 | .26 |
| **iPhone 11** | **68** | **35.7** | **12.8** | **42.0** | **12.5** | **6.3** | **15.1** | **0.50** | **0.1** - **5.1** | **.005** | **.03** |
| iPhone 11 Pro | 27 | 39.6 | 14.0 | 41.7 | 12.6 | 2.1 | 5.0 | 0.16 | 0.0 - 2.6 | .08 | .18 |
| Samsung Galaxy S7 | 20 | 41.1 | 12.0 | 41.6 | 12.6 | 0.5 | 1.2 | 0.04 | 0.1 - 4.0 | .77 | .93 |
| Samsung Galaxy S9 | 20 | 42.2 | 11.7 | 41.6 | 12.6 | 0.5 | 1.3 | 0.04 | 0.1 - 5.2 | .82 | .93 |
| **SBT, sway path, m/s^2^** |  |  |  |  |  |  |  |  |  |  |  |
| iPhone 6 | 76 | 29.3 | 20.5 | 28.3 | 20.7 | 1.0 | 3.6 | 0.05 | 0.1 - 6.1 | .71 | .83 |
| iPhone 6s | 118 | 31.0 | 22.6 | 28.0 | 20.5 | 2.9 | 10.4 | 0.14 | 0.1 - 5.4 | .23 | .65 |
| iPhone 6s Plus | 24 | 31.3 | 25.3 | 28.3 | 20.6 | 3.1 | 10.9 | 0.15 | 0.1 - 4.5 | .12 | .43 |
| iPhone SE | 56 | 25.3 | 17.0 | 28.5 | 20.8 | 3.2 | 11.2 | 0.15 | 0.1 - 9.6 | .46 | .68 |
| iPhone 7 | 189 | 25.9 | 19.2 | 28.8 | 20.9 | 2.9 | 10.0 | 0.14 | 0.1 - 3.7 | .08 | .38 |
| iPhone 7 Plus | 66 | 23.3 | 15.8 | 28.6 | 20.9 | 5.4 | 18.8 | 0.26 | 0.1 - 5.8 | .04 | .27 |
| iPhone 8 | 211 | 29.1 | 20.6 | 28.1 | 20.7 | 1.0 | 3.6 | 0.05 | 0.0 - 3.5 | .53 | .68 |
| iPhone 8 Plus | 86 | 33.0 | 26.0 | 28.0 | 20.2 | 5.0 | 17.9 | 0.24 | 0.1 - 5.2 | .03 | .27 |
| iPhone X Global | 37 | 27.8 | 16.3 | 28.3 | 20.8 | 0.6 | 1.9 | 0.03 | 0.1 - 6.4 | .85 | .85 |
| iPhone X GSM | 49 | 30.6 | 23.3 | 28.2 | 20.6 | 2.4 | 8.5 | 0.12 | 0.1 - 6.7 | .43 | .68 |
| iPhone XS | 49 | 25.5 | 18.7 | 28.5 | 20.8 | 3.0 | 10.4 | 0.14 | 0.1 - 7.8 | .40 | .68 |
| iPhone XS Max Global | 26 | 28.0 | 19.1 | 28.3 | 20.7 | 0.4 | 1.3 | 0.02 | 0.1 - 4.5 | .85 | .85 |
| iPhone XR | 114 | 30.2 | 21.6 | 28.1 | 20.6 | 2.0 | 7.2 | 0.10 | 0.1 - 6.7 | .50 | .68 |
| iPhone 11 | 62 | 24.8 | 18.8 | 28.5 | 20.8 | 3.7 | 13.0 | 0.18 | 0.1 - 9.3 | .37 | .68 |
| **UTT, turn speed, rad/s** |  |  |  |  |  |  |  |  |  |  |  |
| iPhone 6 | 57 | 1.60 | 0.34 | 1.60 | 0.35 | 0.00 | 0.0 | 0.00 | 0.00 - 0.11 | .99 | .99 |
| iPhone 6s | 76 | 1.57 | 0.35 | 1.60 | 0.35 | 0.03 | 1.9 | 0.09 | 0.00 - 0.11 | .52 | .88 |
| iPhone SE | 41 | 1.57 | 0.32 | 1.60 | 0.35 | 0.03 | 1.9 | 0.07 | 0.00 - 0.10 | .54 | .88 |
| iPhone 7 | 151 | 1.61 | 0.36 | 1.59 | 0.35 | 0.02 | 1.3 | 0.06 | 0.00 - 0.07 | .51 | .88 |
| iPhone 7 Plus | 44 | 1.51 | 0.32 | 1.60 | 0.35 | 0.09 | 5.6 | 0.27 | 0.00 - 0.12 | .08 | .55 |
| iPhone 8 | 145 | 1.64 | 0.40 | 1.59 | 0.34 | 0.05 | 3.2 | 0.14 | 0.00 - 0.07 | .13 | .58 |
| iPhone 8 Plus | 69 | 1.53 | 0.33 | 1.60 | 0.35 | 0.08 | 5.0 | 0.22 | 0.00 - 0.10 | .08 | .54 |
| iPhone X Global | 25 | 1.59 | 0.24 | 1.60 | 0.35 | 0.00 | 0.0 | 0.01 | 0.00 - 0.12 | .96 | .99 |
| iPhone X GSM | 44 | 1.66 | 0.33 | 1.59 | 0.35 | 0.07 | 4.4 | 0.20 | 0.00 - 0.12 | .20 | .65 |
| iPhone XS | 34 | 1.58 | 0.33 | 1.60 | 0.35 | 0.01 | 0.6 | 0.04 | 0.00 - 0.16 | .83 | .99 |
| iPhone XS Max Global | 21 | 1.63 | 0.39 | 1.60 | 0.35 | 0.03 | 1.9 | 0.09 | 0.00 - 0.09 | .46 | .88 |
| iPhone XR | 81 | 1.60 | 0.37 | 1.60 | 0.35 | 0.00 | 0.0 | 0.01 | 0.00 - 0.14 | .98 | .99 |
| iPhone 11 | 54 | 1.60 | 0.28 | 1.60 | 0.35 | 0.01 | 0.6 | 0.02 | 0.00 - 0.18 | .93 | .98 |
| **2WMT, steps, n** |  |  |  |  |  |  |  |  |  |  |  |
| iPhone 6 | 38 | 193.2 | 29.7 | 197.5 | 38.0 | 4.3 | 2.2 | 0.12 | 0.2 - 14.2 | .48 | .94 |
| iPhone 6s | 64 | 192.7 | 41.6 | 197.8 | 36.9 | 5.1 | 2.6 | 0.14 | 0.2 - 14.4 | .42 | .94 |
| iPhone SE | 29 | 200.1 | 36.9 | 197.0 | 37.5 | 3.1 | 1.6 | 0.08 | 0.2 - 11.3 | .54 | .94 |
| iPhone 7 | 86 | 203.4 | 46.0 | 195.9 | 35.4 | 7.5 | 3.8 | 0.20 | 0.1 - 10.2 | .09 | .94 |
| iPhone 7 Plus | 31 | 198.2 | 30.7 | 197.1 | 37.9 | 1.1 | 0.6 | 0.03 | 0.2 - 15.8 | .87 | .99 |
| iPhone 8 | 82 | 197.0 | 34.5 | 197.2 | 38.1 | 0.1 | 0.1 | 0.00 | 0.1 - 10.0 | .97 | .99 |
| iPhone 8 Plus | 45 | 194.2 | 37.2 | 197.5 | 37.5 | 3.3 | 1.7 | 0.09 | 0.2 - 13.4 | .56 | .94 |
| iPhone X GSM | 22 | 188.1 | 40.4 | 197.6 | 37.3 | 9.4 | 4.8 | 0.25 | 0.2 - 16.1 | .18 | .94 |
| iPhone XS | 20 | 196.5 | 29.9 | 197.2 | 37.8 | 0.7 | 0.4 | 0.02 | 0.2 - 18.7 | .93 | .99 |
| iPhone XR | 54 | 199.7 | 36.4 | 196.9 | 37.6 | 2.8 | 1.4 | 0.08 | 0.2 - 12.0 | .60 | .94 |
| iPhone 11 | 39 | 197.3 | 33.1 | 197.2 | 37.9 | 0.1 | 0.1 | 0.00 | 0.2 - 19.4 | .99 | .99 |

^a^All other device models pooled together.
^b^*P*-values were adjusted for multiple comparisons with false discovery rate (FDR) correction using the Benjamini-Hochberg method.
2MWT, Two-Minute Walk Test; IPS, Information Processing Speed; IPS DD, Information Processing Speed Digit–Digit; PT, Pinching Test; SBT, Static Balance Test; SD, standard deviation; UTT, U-Turn Test.

**Table S2. Absolute and percent median differences across OS platforms (sensitivity analysis).**

| **Active tests** | **iOS** | | **Android** | | **Median difference from Android** | | **Permutation test** | |
| --- | --- | --- | --- | --- | --- | --- | --- | --- |
|  | **n** | **Median** | **n** | **Median** | **Absolute difference** | **Percent difference** | **95% CI** | ***P*_unadjusted_** |
| IPS, correct responses, n | 734 | 64.3 | 341 | 63.1 | 1.2 | 1.8 | 0.0 - 1.4 | .08 |
| IPS DD, correct responses, n | 714 | 24.7 | 333 | 24.6 | 0.1 | 0.6 | 0.0 - 0.4 | .38 |
| **PT, successful pinches, n** | **1233** | **42.3** | **338** | **38.5** | **3.8** | **10.0** | **0.0** - **2.3** | **<.001** |
| SBT, sway path, m/s2 | 1313 | 19.7 | 126 | 20.7 | 1.0 | 4.8 | 0.0 - 3.2 | .46 |
| UTT, turn speed, rad/s | 975 | 1.56 | 219 | 1.58 | 0.02 | 1.3 | 0.00 - 0.06 | .42 |
| 2WMT, steps, n | 619 | 200.3 | 168 | 197.0 | 3.4 | 1.7 | 0.1 - 8.5 | .35 |

Entries highlighted in bold are statistically significant (*P*<.05).
2MWT, Two-Minute Walk Test; IPS, Information Processing Speed; IPS DD, Information Processing Speed Digit–Digit; PT, Pinching Test; SBT, Static Balance Test; UTT, U-Turn Test.

**Table S3. Absolute and percent median differences by iOS version (sensitivity analysis).**

| **Active test** | **iOS version subgroup** | | **Reference group^a^ median** | **Median difference from reference group** | | **Permutation test** | | |
| --- | --- | --- | --- | --- | --- | --- | --- | --- |
|  | **n** | **Median** |  | **Absolute difference** | **Percent difference** | **95% CI** | ***P*_unadjusted_** | ***P*_adjusted_^b^** |
| **IPS, correct responses, n** |  |  |  |  |  |  |  |  |
| iOS 11 | 84 | 65.0 | 64.3 | 0.7 | 1.1 | 0.0 - 2.7 | .58 | .74 |
| iOS 12 | 304 | 63.3 | 64.9 | 1.6 | 2.5 | 0.0 - 1.7 | .03 | .16 |
| iOS 13 | 199 | 65.0 | 63.9 | 1.0 | 1.6 | 0.0 - 2.0 | .27 | .67 |
| iOS 14 | 118 | 64.9 | 64.3 | 0.6 | 1.0 | 0.0 - 2.5 | .59 | .74 |
| iOS 15 | 23 | 63.9 | 64.3 | 0.4 | 0.6 | 0.1 - 5.1 | .89 | .89 |
| **IPS DD, correct responses, n** |  |  |  |  |  |  |  |  |
| iOS 11 | 83 | 25.0 | 24.7 | 0.3 | 1.3 | 0.0 - 0.8 | .27 | .27 |
| **iOS 12** | **296** | **24.3** | **25.0** | **0.7** | **2.8** | **0.0** - **0.5** | **.001** | **.007** |
| iOS 13 | 190 | 24.9 | 24.6 | 0.4 | 1.5 | 0.0 - 0.6 | .19 | .24 |
| iOS 14 | 117 | 25.0 | 24.6 | 0.4 | 1.7 | 0.0 - 0.7 | .18 | .24 |
| iOS 15 | 23 | 25.6 | 24.7 | 0.9 | 3.5 | 0.0 - 1.4 | .17 | .24 |
| **PT, successful pinches, n** |  |  |  |  |  |  |  |  |
| iOS 11 | 38 | 41.2 | 42.3 | 1.1 | 2.6 | 0.1 - 5.6 | .67 | .94 |
| iOS 12 | 568 | 42.3 | 42.3 | 0.0 | 0.1 | 0.1 - 2.0 | .99 | .99 |
| iOS 13 | 399 | 42.9 | 42.1 | 0.9 | 2.0 | 0.0 - 2.1 | .36 | .94 |
| iOS 14 | 185 | 41.5 | 42.5 | 0.9 | 2.2 | 0.0 - 2.7 | .46 | .94 |
| iOS 15 | 38 | 41.5 | 42.3 | 0.8 | 1.9 | 0.1 - 5.6 | .75 | .94 |
| **SBT, sway path, m/s2** |  |  |  |  |  |  |  |  |
| iOS 11 | 181 | 20.2 | 19.7 | 0.5 | 2.7 | 0.0 - 2.8 | .64 | .94 |
| iOS 12 | 578 | 20.5 | 19.0 | 1.5 | 7.7 | 0.0 - 1.7 | .06 | .16 |
| iOS 13 | 348 | 18.2 | 20.4 | 2.2 | 10.6 | 0.0 - 2.2 | .03 | .14 |
| iOS 14 | 163 | 19.7 | 19.7 | 0.0 | 0.2 | 0.0 - 2.9 | .94 | .94 |
| iOS 15 | 38 | 19.4 | 19.7 | 0.3 | 1.6 | 0.1 - 6.5 | .89 | .94 |
| **UTT, turn speed, rad/s** |  |  |  |  |  |  |  |  |
| iOS 11 | 121 | 1.52 | 1.56 | 0.04 | 2.2 | 0.00 - 0.09 | .33 | .55 |
| iOS 12 | 409 | 1.53 | 1.56 | 0.03 | 2.0 | 0.00 - 0.05 | .20 | .51 |
| iOS 13 | 279 | 1.59 | 1.54 | 0.05 | 2.9 | 0.00 - 0.06 | .08 | .41 |
| iOS 14 | 134 | 1.56 | 1.55 | 0.01 | 0.6 | 0.00 - 0.08 | .79 | .79 |
| iOS 15 | 29 | 1.61 | 1.56 | 0.05 | 3.3 | 0.00 - 0.17 | .48 | .60 |
| **2WMT, steps, n** |  |  |  |  |  |  |  |  |
| iOS 11 | 67 | 207.6 | 198.7 | 8.8 | 4.5 | 0.4 - 12.0 | .12 | .16 |
| iOS 12 | 241 | 194.7 | 203.2 | 8.5 | 4.2 | 0.3 - 8.0 | .02 | .09 |
| iOS 13 | 179 | 200.3 | 200.3 | 0.1 | 0.0 | 0.3 - 8.3 | 1.0 | 1.0 |
| iOS 14 | 96 | 208.1 | 199.0 | 9.1 | 4.6 | 0.2 - 10.3 | .059 | .15 |
| iOS 15 | 32 | 189.8 | 201.1 | 11.3 | 5.6 | 0.3 - 16.8 | .13 | .16 |

Entries highlighted in bold are statistically significant according to their adjusted *P*-values (*P* adjusted <.05).
^a^All other iOS versions pooled together.
^b^*P*-values were adjusted for multiple comparisons with false discovery rate (FDR) correction using the Benjamini-Hochberg method. Entries highlighted in bold are statistically significant according to the adjusted *P*-values.
2MWT, Two-Minute Walk Test; IPS, Information Processing Speed; IPS DD, Information Processing Speed Digit–Digit; PT, Pinching Test; SBT, Static Balance Test; UTT, U-Turn Test.

**Table S4. Absolute and percent median differences by Android version (sensitivity analysis).**

| **Active test** | **Android version subgroup** | | **Reference group^a^ median** | **Median difference from reference group** | | **Permutation test** | | |
| --- | --- | --- | --- | --- | --- | --- | --- | --- |
|  | **n** | **Median** |  | **Absolute difference** | **Percent difference** | **95% CI** | ***P*_unadjusted_** | ***P*_adjusted_^b^** |
| **IPS, correct responses, n** |  |  |  |  |  |  |  |  |
| Android 8 | 62 | 62.8 | 63.4 | 0.6 | 1.0 | 0.1 - 3.5 | .76 | .76 |
| Android 9 | 114 | 62.8 | 63.4 | 0.6 | 0.9 | 0.1 - 3.1 | .74 | .76 |
| Android 10 | 75 | 63.5 | 62.8 | 0.7 | 1.1 | 0.1 - 3.2 | .72 | .76 |
| **IPS DD, correct responses, n** |  |  |  |  |  |  |  |  |
| Android 8 | 62 | 24.6 | 24.6 | 0.0 | 0.0 | 0.0 - 1.1 | .99 | .99 |
| Android 9 | 109 | 25.1 | 24.5 | 0.6 | 2.6 | 0.0 - 0.9 | .13 | .38 |
| Android 10 | 74 | 24.4 | 24.6 | 0.2 | 0.7 | 0.0 - 1.0 | .71 | .99 |
| **PT, successful pinches, n** |  |  |  |  |  |  |  |  |
| Android 8 | 61 | 41.0 | 38.0 | 3.0 | 8.0 | 0.4 - 6.2 | .21 | .32 |
| Android 9 | 113 | 37.5 | 40.3 | 2.8 | 7.0 | 0.4 - 5.0 | .16 | .32 |
| Android 10 | 74 | 38.4 | 39.0 | 0.5 | 1.4 | 0.1 - 5.5 | .86 | .86 |
| **SBT, sway path, m/s2** |  |  |  |  |  |  |  |  |
| Android 8 | 23 | 16.9 | 20.3 | 3.5 | 17.0 | 0.1 - 8.6 | .45 | .68 |
| Android 9 | 38 | 22.0 | 18.3 | 3.7 | 19.9 | 0.1 - 7.3 | .34 | .68 |
| Android 10 | 24 | 18.4 | 20.2 | 1.8 | 8.7 | 0.1 - 8.3 | .70 | .70 |
| **UTT, turn speed, rad/s** |  |  |  |  |  |  |  |  |
| Android 8 | 38 | 1.56 | 1.59 | 0.02 | 1.3 | 0.00 - 0.13 | .69 | .69 |
| Android 9 | 72 | 1.61 | 1.55 | 0.06 | 3.9 | 0.00 - 0.10 | .21 | .46 |
| Android 10 | 55 | 1.55 | 1.60 | 0.05 | 3.1 | 0.00 - 0.11 | .31 | .46 |
| **2WMT, steps, n** |  |  |  |  |  |  |  |  |
| Android 8 | 33 | 193.2 | 197.6 | 4.4 | 2.2 | 0.2 - 18.7 | .69 | .69 |
| Android 9 | 57 | 203.4 | 193.5 | 9.9 | 5.1 | 0.2 - 14.5 | .17 | .50 |
| Android 10 | 39 | 193.8 | 200.5 | 6.7 | 3.3 | 0.2 - 16.8 | .48 | .69 |

^a^All other Android versions pooled together.
^b^*P*-values were adjusted for multiple comparisons with false discovery rate (FDR) correction using the Benjamini-Hochberg method.
2MWT, Two-Minute Walk Test; IPS, Information Processing Speed; IPS DD, Information Processing Speed Digit–Digit; PT, Pinching Test; SBT, Static Balance Test; UTT, U-Turn Test.

**Table S5. Absolute and percent median differences by device model (sensitivity analysis).**

| **Active test** | **Device model subgroup** | | **Reference group^a^ median** | **Median difference from reference group** | | **Permutation test** | | |
| --- | --- | --- | --- | --- | --- | --- | --- | --- |
|  | **n** | **Median** |  | **Absolute difference** | **Percent difference** | **95% CI** | ***P*_unadjusted_** | ***P*_adjusted_^b^** |
| **IPS, correct responses, n** |  |  |  |  |  |  |  |  |
| iPhone 6 | 45 | 64.9 | 64.2 | 0.7 | 1.2 | 0.1 - 5.3 | .76 | .96 |
| iPhone 6s | 70 | 63.3 | 64.3 | 1.0 | 1.6 | 0.1 - 5.3 | .68 | .96 |
| iPhone SE | 36 | 65.9 | 64.0 | 1.9 | 3.0 | 0.1 - 4.0 | .29 | .96 |
| iPhone 7 | 98 | 64.3 | 64.2 | 0.1 | 0.1 | 0.0 - 3.7 | .96 | .96 |
| iPhone 7 Plus | 33 | 65.0 | 64.2 | 0.8 | 1.3 | 0.0 - 3.1 | .55 | .96 |
| iPhone 8 | 108 | 64.1 | 64.2 | 0.2 | 0.2 | 0.0 - 2.7 | .90 | .96 |
| iPhone 8 Plus | 62 | 65.3 | 64.2 | 1.1 | 1.7 | 0.0 - 4.3 | .58 | .96 |
| iPhone X GSM | 31 | 64.8 | 64.2 | 0.6 | 0.9 | 0.0 - 2.7 | .65 | .96 |
| iPhone XS | 20 | 60.4 | 64.4 | 4.0 | 6.2 | 0.1 - 3.2 | .004 | .056 |
| iPhone XR | 68 | 64.8 | 64.1 | 0.6 | 1.0 | 0.1 - 4.1 | .74 | .96 |
| iPhone 11 | 38 | 64.7 | 64.2 | 0.5 | 0.8 | 0.0 - 4.4 | .82 | .96 |
| Samsung Galaxy S7 | 21 | 64.4 | 64.3 | 1.8 | 2.9 | 0.0 - 3.1 | .20 | .96 |
| Samsung Galaxy S9 | 20 | 62.5 | 64.2 | 1.8 | 2.8 | 0.1 - 5.2 | .46 | .96 |
| **IPS DD, correct responses, n** |  |  |  |  |  |  |  |  |
| iPhone 6 | 44 | 24.8 | 24.6 | 0.3 | 1.1 | 0.0 - 1.6 | .71 | .84 |
| iPhone 6s | 68 | 24.4 | 24.7 | 0.3 | 1.1 | 0.0 - 1.1 | .57 | .84 |
| iPhone SE | 35 | 24.0 | 24.7 | 0.7 | 2.8 | 0.0 - 1.1 | .21 | .59 |
| iPhone 7 | 96 | 24.5 | 24.7 | 0.2 | 0.8 | 0.0 - 0.9 | .65 | .84 |
| iPhone 7 Plus | 34 | 25.1 | 24.6 | 0.5 | 2.0 | 0.0 - 0.8 | .15 | .59 |
| iPhone 8 | 101 | 24.7 | 24.6 | 0.1 | 0.5 | 0.0 - 1.2 | .84 | .84 |
| iPhone 8 Plus | 59 | 24.8 | 24.6 | 0.3 | 1.1 | 0.0 - 0.8 | .50 | .84 |
| iPhone X GSM | 31 | 25.1 | 24.6 | 0.6 | 2.2 | 0.0 - 1.0 | .25 | .59 |
| iPhone XS | 20 | 23.6 | 24.7 | 1.1 | 4.6 | 0.0 - 1.3 | .04 | .36 |
| iPhone XR | 65 | 24.4 | 24.6 | 0.2 | 0.9 | 0.0 - 1.3 | .71 | .84 |
| iPhone 11 | 39 | 25.4 | 24.6 | 0.8 | 3.4 | 0.0 - 1.0 | .059 | .36 |
| Samsung Galaxy S7 | 21 | 24.4 | 4.6 | 0.2 | 0.7 | 0.0 - 1.6 | .80 | .84 |
| **PT, successful pinches, n** |  |  |  |  |  |  |  |  |
| iPhone 6 | 55 | 39.5 | 43.0 | 3.6 | 8.3 | 0.1 - 7.4 | .27 | .49 |
| iPhone 6s | 95 | 43.6 | 42.8 | 0.8 | 1.9 | 0.1 - 7.6 | .81 | .93 |
| **iPhone SE** | **46** | **37.6** | **43.0** | **5.4** | **12.6** | **0.1** - **4.2** | **.004** | **.03** |
| iPhone 7 | 156 | 44.6 | 42.4 | 2.1 | 5.0 | 0.1 - 6.7 | .46 | .57 |
| iPhone 7 Plus | 47 | 39.4 | 42.9 | 3.6 | 8.3 | 0.1 - 4.6 | .09 | .28 |
| iPhone 8 | 221 | 42.7 | 42.8 | 0.2 | 0.4 | 0.0 - 3.7 | .94 | .94 |
| iPhone 8 Plus | 85 | 44.2 | 42.5 | 1.7 | 4.0 | 0.1 - 2.9 | .20 | .40 |
| iPhone X Global | 32 | 45.9 | 42.5 | 3.4 | 8.0 | 0.1 - 5.0 | .13 | .33 |
| **iPhone X GSM** | **53** | **47.7** | **42.4** | **5.4** | **12.7** | **0.0** - **2.6** | **<.001** | **<.001** |
| iPhone XS | 51 | 45.8 | 42.7 | 3.1 | 7.3 | 0.1 - 3.9 | .07 | .26 |
| iPhone XS Max Global | 32 | 44.6 | 42.8 | 1.8 | 4.2 | 0.1 - 5.0 | .43 | .57 |
| iPhone XR | 132 | 40.9 | 43.0 | 2.1 | 4.9 | 0.1 - 4.8 | .31 | .50 |
| iPhone 11 | 68 | 37.1 | 43.2 | 6.1 | 14.0 | 0.1 - 6.1 | .03 | .14 |
| iPhone 11 Pro | 27 | 40.9 | 42.9 | 2.0 | 4.7 | 0.1 - 3.1 | .14 | .33 |
| Samsung Galaxy S7 | 20 | 43.0 | 42.8 | 0.2 | 0.4 | 0.1 - 4.7 | .94 | .94 |
| Samsung Galaxy S9 | 20 | 44.8 | 42.8 | 2.1 | 4.8 | 0.1 - 5.9 | .44 | .57 |
| **SBT, sway path, m/s2** |  |  |  |  |  |  |  |  |
| iPhone 6 | 76 | 20.9 | 19.7 | 1.3 | 6.4 | 0.1 - 4.9 | .52 | .80 |
| iPhone 6s | 118 | 21.7 | 19.7 | 2.1 | 10.4 | 0.0 - 4.5 | .28 | .74 |
| iPhone 6s Plus | 24 | 18.9 | 19.8 | 0.9 | 4.5 | 0.0 - 3.5 | .51 | .80 |
| iPhone SE | 56 | 19.5 | 19.7 | 0.2 | 1.0 | 0.1 - 9.8 | .95 | .95 |
| iPhone 7 | 189 | 18.3 | 20.3 | 2.1 | 10.2 | 0.0 - 2.8 | .11 | .74 |
| iPhone 7 Plus | 66 | 17.0 | 20.0 | 2.9 | 14.7 | 0.1 - 4.8 | .13 | .74 |
| iPhone 8 | 221 | 19.7 | 19.8 | 0.1 | 0.7 | 0.0 - 2.7 | .87 | .93 |
| iPhone 8 Plus | 86 | 21.3 | 19.7 | 1.6 | 8.3 | 0.0 - 4.2 | .35 | .74 |
| iPhone X Global | 37 | 21.6 | 19.7 | 1.9 | 9.8 | 0.1 - 5.1 | .37 | .74 |
| iPhone X GSM | 49 | 20.2 | 19.7 | 0.5 | 2.5 | 0.1 - 5.7 | .82 | .93 |
| iPhone XS | 49 | 19.2 | 19.8 | 0.5 | 2.6 | 0.1 - 6.8 | .84 | .93 |
| iPhone XS Max Global | 26 | 20.2 | 19.7 | 0.5 | 2.5 | 0.1 - 3.5 | .74 | .93 |
| iPhone XR | 114 | 22.1 | 19.6 | 2.6 | 13.1 | 0.1 - 5.7 | .26 | .74 |
| iPhone 11 | 62 | 15.8 | 19.8 | 4.1 | 20.5 | 0.1 - 9.1 | .18 | .74 |
| **UTT, turn speed, rad/s** |  |  |  |  |  |  |  |  |
| iPhone 6 | 57 | 1.51 | 1.56 | 0.05 | 3.2 | 0.00 - 0.13 | .35 | .88 |
| iPhone 6s | 76 | 1.57 | 1.56 | 0.01 | 0.6 | 0.00 - 0.12 | .82 | .89 |
| iPhone SE | 41 | 1.56 | 1.56 | 0.00 | 0.0 | 0.00 - 0.11 | .96 | .96 |
| iPhone 7 | 151 | 1.57 | 1.55 | 0.02 | 1.3 | 0.00 - 0.08 | .52 | .88 |
| iPhone 7 Plus | 44 | 1.47 | 1.56 | 0.09 | 5.8 | 0.00 - 0.14 | .14 | .59 |
| iPhone 8 | 145 | 1.58 | 1.56 | 0.02 | 1.3 | 0.00 - 0.08 | .50 | .88 |
| iPhone 8 Plus | 69 | 1.49 | 1.57 | 0.08 | 5.1 | 0.00 - 0.11 | .13 | .59 |
| iPhone X Global | 25 | 1.61 | 1.56 | 0.05 | 3.2 | 0.00 - 0.15 | .40 | .88 |
| iPhone X GSM | 44 | 1.65 | 1.56 | 0.09 | 5.8 | 0.00 - 0.14 | .13 | .59 |
| iPhone XS | 34 | 1.54 | 1.56 | 0.02 | 1.3 | 0.00 - 0.19 | .78 | .89 |
| iPhone XS Max Global | 21 | 1.58 | 1.56 | 0.02 | 1.3 | 0.00 - 0.11 | .57 | .88 |
| iPhone XR | 81 | 1.58 | 1.56 | 0.03 | 1.9 | 0.00 - 0.16 | .68 | .88 |
| iPhone 11 | 54 | 1.59 | 1.56 | 0.04 | 2.6 | 0.00 - 0.21 | .65 | .88 |
| **2WMT, steps, n** |  |  |  |  |  |  |  |  |
| iPhone 6 | 38 | 193.0 | 201.9 | 8.8 | 4.4 | 0.1 - 14.1 | .20 | .78 |
| iPhone 6s | 64 | 203.6 | 201.6 | 2.0 | 1.0 | 0.2 - 14.0 | .77 | .87 |
| iPhone SE | 29 | 210.6 | 201.4 | 9.2 | 4.6 | 0.2 - 11.8 | .09 | .78 |
| iPhone 7 | 86 | 204.2 | 201.4 | 2.8 | 1.4 | 0.2 - 10.8 | .55 | .87 |
| iPhone 7 Plus | 31 | 195.4 | 202.0 | 6.6 | 3.3 | 0.1 - 15.5 | .36 | .87 |
| iPhone 8 | 82 | 201.4 | 201.7 | 0.3 | 0.2 | 0.1 - 10.8 | .93 | .93 |
| iPhone 8 Plus | 45 | 200.0 | 201.8 | 1.8 | 0.9 | 0.1 - 13.0 | .79 | .87 |
| iPhone X GSM | 22 | 192.5 | 202.0 | 9.5 | 4.7 | 0.1 - 15.8 | .21 | .78 |
| iPhone XS | 20 | 198.3 | 201.7 | 3.4 | 1.7 | 0.3 - 19.1 | .70 | .87 |
| iPhone XR | 54 | 206.1 | 201.4 | 4.7 | 2.3 | 0.2 - 12.4 | .42 | .87 |
| iPhone 11 | 39 | 204.3 | 201.4 | 2.9 | 1.5 | 0.3 - 19.8 | .74 | .87 |

Entries highlighted in bold are statistically significant according to their adjusted *P*-value (*P* adjusted <.05). ^a^All other device models pooled together.
^b^*P*-values were adjusted for multiple comparisons with false discovery rate (FDR) correction using the Benjamini-Hochberg method.
2MWT, Two-Minute Walk Test; IPS, Information Processing Speed; IPS DD, Information Processing Speed Digit–Digit; PT, Pinching Test; SBT, Static Balance Test; UTT, U-Turn Test.


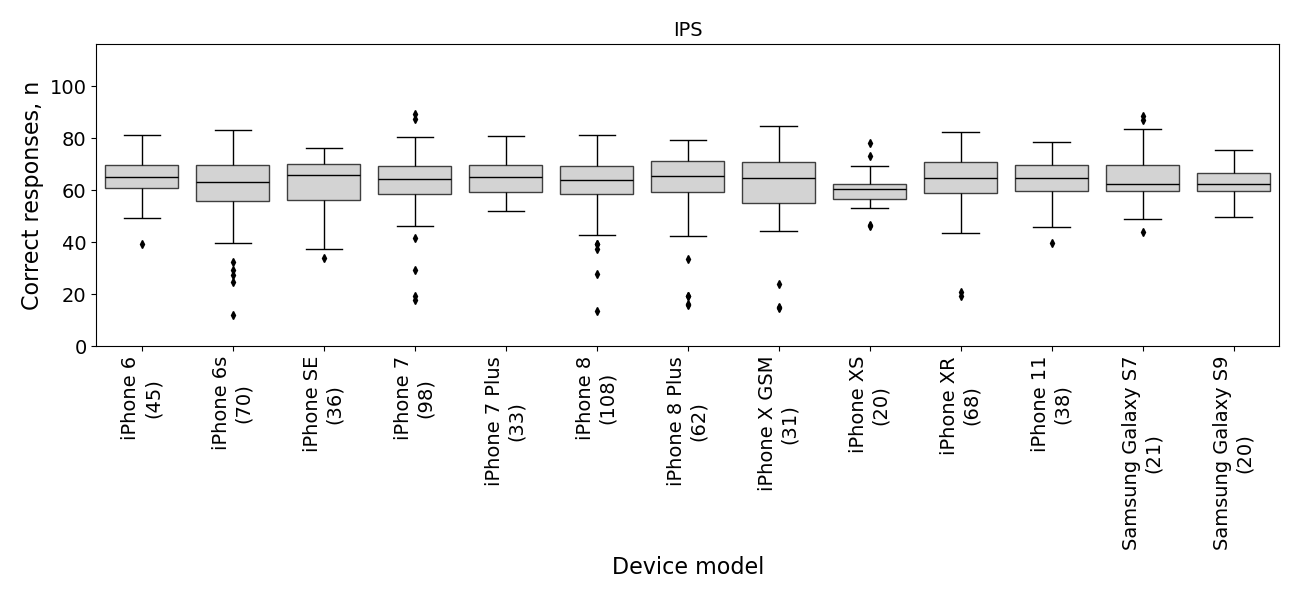

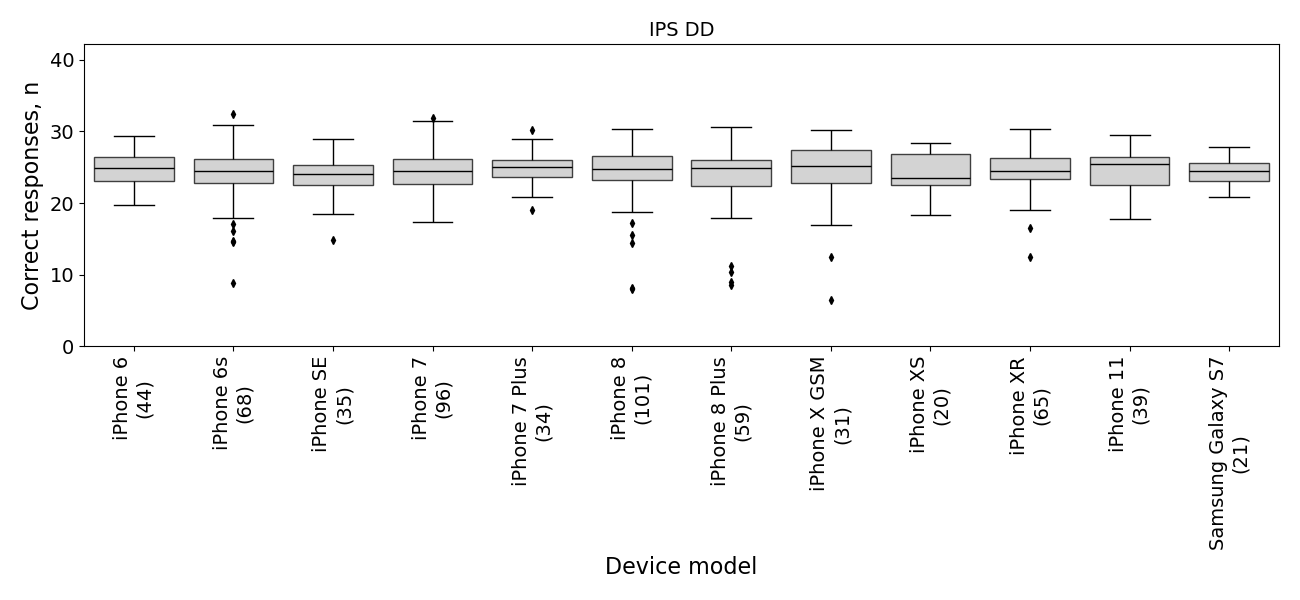

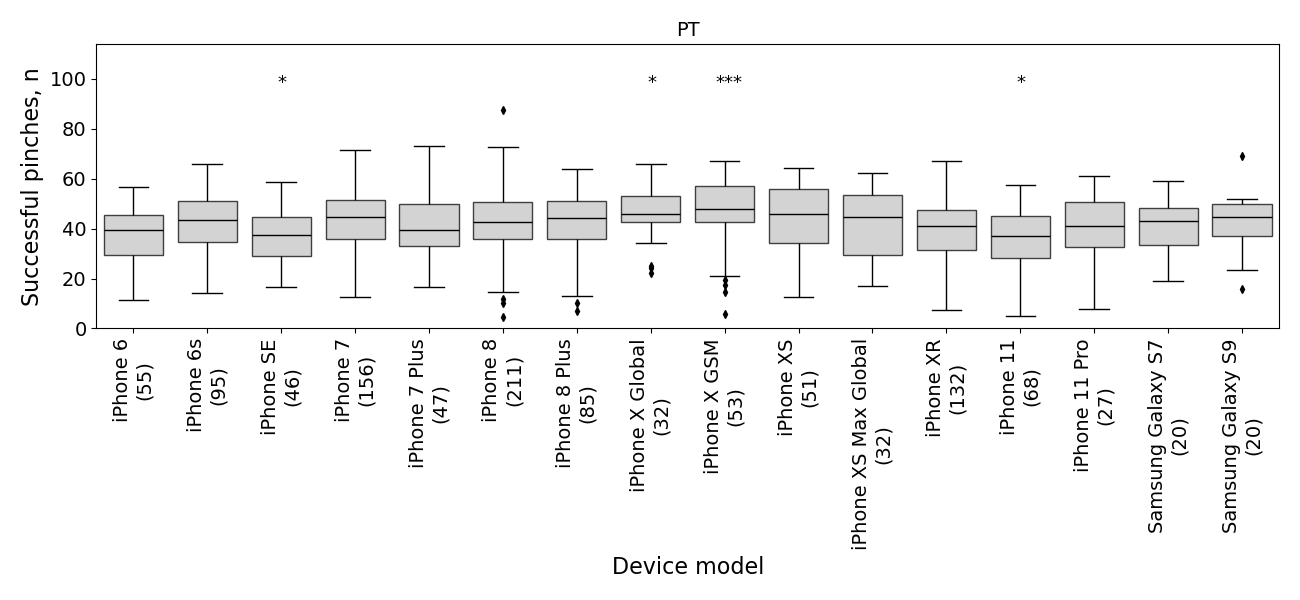

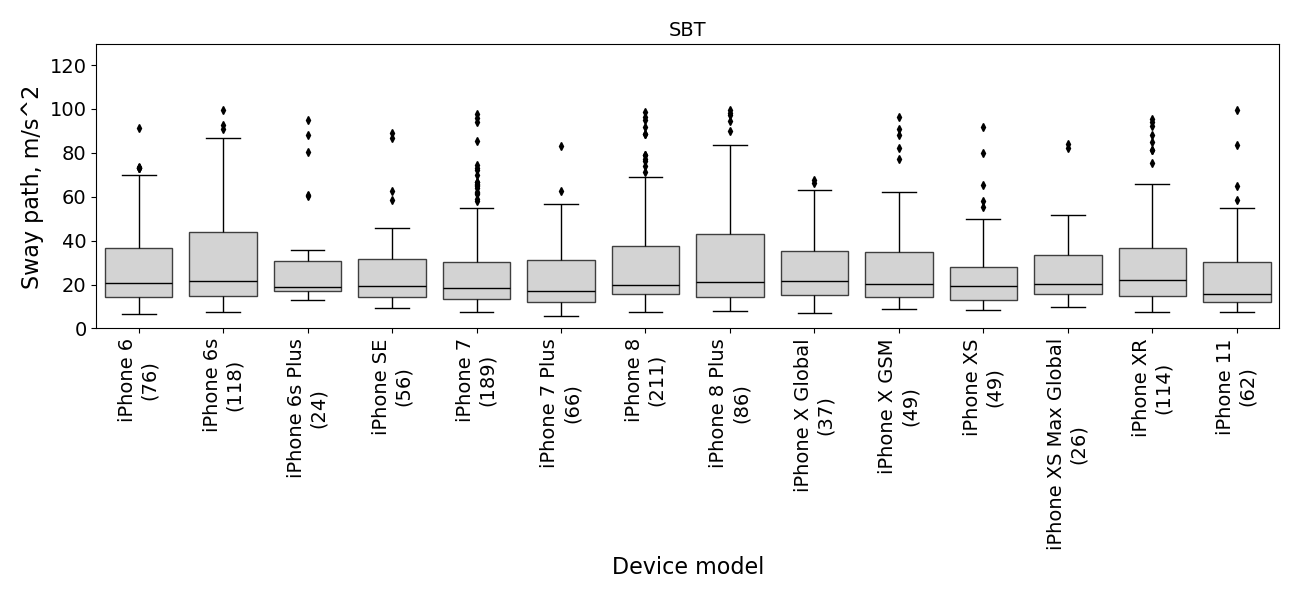

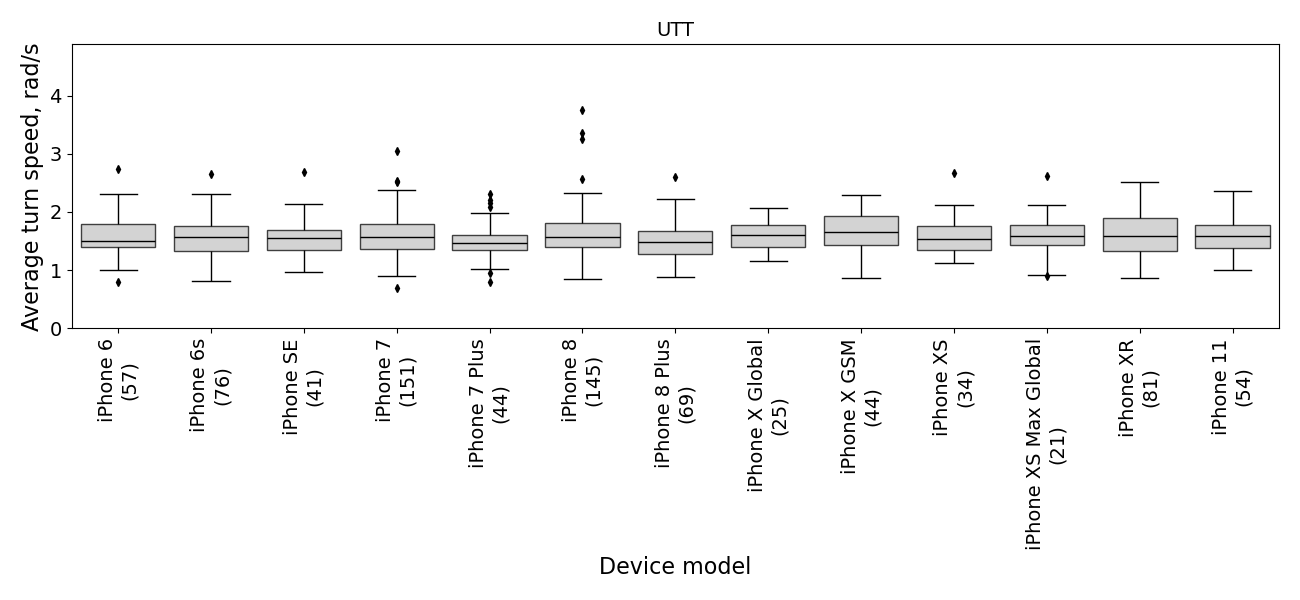

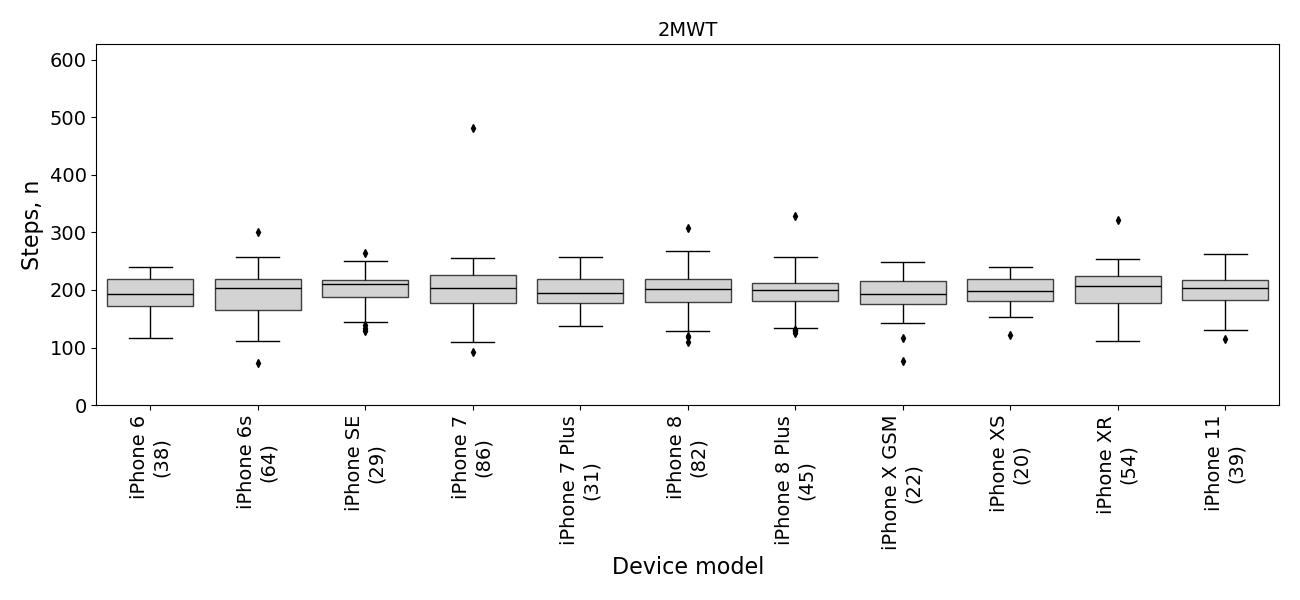


**Figure S1.** Measurement equivalence by device model. No evidence of a systematic lack of equivalence was found as most device models did not show a statistically significant difference from the overall group mean (i.e., all device models combined). Permutation testing revealed no statistically significant differences after correcting for multiple comparisons. Absolute and percent differences, effect sizes and exact *P*-values (unadjusted and adjusted) are reported in Table S1. Brackets indicate the sample size. **P*<.05 and ****P*<.001 after adjusting for multiple comparisons.
2MWT, Two-Minute Walk Test; IQR, interquartile range; IPS, Information Processing Speed; IPS DD, Information Processing Speed Digit–Digit; PT, Pinching Test; SBT, Static Balance Test; UTT, U-Turn Test.
